# Improved memory CD8 T cell response to delayed vaccine boost is associated with a distinct molecular signature

**DOI:** 10.3389/fimmu.2023.1043631

**Published:** 2023-02-14

**Authors:** Ambra Natalini, Sonia Simonetti, Gabriele Favaretto, Lorenzo Lucantonio, Giovanna Peruzzi, Miguel Muñoz-Ruiz, Gavin Kelly, Alessandra M. Contino, Roberta Sbrocchi, Simone Battella, Stefania Capone, Antonella Folgori, Alfredo Nicosia, Angela Santoni, Adrian C. Hayday, Francesca Di Rosa

**Affiliations:** ^1^ Institute of Molecular Biology and Pathology, National Research Council of Italy (CNR), Rome, Italy; ^2^ Department of Molecular Medicine, University of Rome “Sapienza”, Rome, Italy; ^3^ Center for Life Nano- & Neuro-Science, Fondazione Istituto Italiano di Tecnologia (IIT), Rome, Italy; ^4^ Immunosurveillance Laboratory, The Francis Crick Institute, London, United Kingdom; ^5^ Bioinformatic and Biostatistics Science and Technology Platform, The Francis Crick Institute, London, United Kingdom; ^6^ ReiThera S.R.L., Rome, Italy; ^7^ CEINGE, Naples, Italy; ^8^ Department of Molecular Medicine and Medical Biotechnology, University of Naples Federico II, Naples, Italy; ^9^ IRCCS Neuromed, Isernia, Italy; ^10^ Peter Gorer Department of Immunobiology, King’s College London, London, United Kingdom; ^11^ National Institute for Health Research (NIHR), Biomedical Research Center (BRC), Guy’s and St Thomas’ NHS Foundation Trust and King’s College London, London, United Kingdom

**Keywords:** CD8 T cells, memory, prime-boost interval, transcriptomic profile, vaccination

## Abstract

Effective secondary response to antigen is a hallmark of immunological memory. However, the extent of memory CD8 T cell response to secondary boost varies at different times after a primary response. Considering the central role of memory CD8 T cells in long-lived protection against viral infections and tumors, a better understanding of the molecular mechanisms underlying the changing responsiveness of these cells to antigenic challenge would be beneficial. We examined here primed CD8 T cell response to boost in a BALB/c mouse model of intramuscular vaccination by priming with HIV-1 gag-encoding Chimpanzee adenovector, and boosting with HIV-1 gag-encoding Modified Vaccinia virus Ankara. We found that boost was more effective at day(d)100 than at d30 post-prime, as evaluated at d45 post-boost by multi-lymphoid organ assessment of gag-specific CD8 T cell frequency, CD62L-expression (as a guide to memory status) and *in vivo* killing. RNA-sequencing of splenic gag-primed CD8 T cells at d100 revealed a quiescent, but highly responsive signature, that trended toward a central memory (CD62L^+^) phenotype. Interestingly, gag-specific CD8 T cell frequency selectively diminished in the blood at d100, relative to the spleen, lymph nodes and bone marrow. These results open the possibility to modify prime/boost intervals to achieve an improved memory CD8 T cell secondary response.

## Introduction

CD8 T cells are one of the pillars of adaptive immunity. Naïve CD8 T cells are primed in lymph nodes (LNs) and the spleen by mature dendritic cells (DCs), which present short antigen (Ag)-derived peptides in the groove of Major Histocompatibility Complex (MHC) class I (MHC-I) molecules, together with sufficient costimulatory signals and in the context of CD4 T cell help. Thereupon, Ag-responding CD8 T cells proliferate and differentiate as effectors and memory cells. In contrast to naïve T cells that mostly recirculate in blood, spleen and LNs, effector and memory T cells have enhanced capacity to migrate to the bone marrow (BM) and extra-lymphoid tissues (1). Upon recognition of Ag-MHC-I presented by target cells, effector CD8 T cells display cytotoxic activity and/or cytokine production, thus critically contributing to the clearance of Ag-expressing cells. Afterwards most effector CD8 T cells die, while a few memory CD8 T cells remain durable, ready to provide an enhanced secondary response in case of subsequent encounter with the same Ag (2).

Although memory CD8 T cells are critical for durable protection against viral infections and tumors (3), some questions of their biology are still unsolved, hampering the possibility to manipulate CD8 T cell responses, for example in vaccine design. Generating CD8 T cell-eliciting vaccines proved challenging for many years. The obstacles have now been reduced by some effective platforms, including those based on adenoviral vectors (4–6) and on mRNA (7). Nevertheless, there is no clear protocol for how best to induce long-lasting immunity in a predictable manner in respect to vaccine dose and route of administration. Similarly, even though it has been suggested that delaying vaccine boost can be beneficial for improved CD8 T cell response, at least to adenoviral vector-based vaccines, there is no current agreement on the criteria for setting the appropriate time intervals between repeated injections (8–10). Solving these issues can be extremely beneficial for immunological understanding and for public health policy, as emphasized by the current COVID-19 pandemic (11–13).

We previously showed that the Chimpanzee adenovector ChAd-gag induced a strong clonal expansion of CD8 T cells against the model antigen Human Immunodeficiency Virus-1 (HIV-1) gag in BALB/c mice (14). Indeed, after intramuscular (i.m.) injection with ChAd-gag, gag-specific CD8 T cells in S-G_2_/M phases of cell cycle were found not only in LNs and spleen, but also in peripheral blood. Results were similar after boosting with Modified Vaccinia virus Ankara (MVA)-gag (14). Using this ChAd-gag/MVA-gag model (14, 15), we have now addressed here the impact of the prime/boost time interval on gag-specific CD8 T cell immunity. Our hypothesis was that establishment and regulation of quiescence in primed CD8 T cells after clonal expansion would be closely related to the cells’ responsiveness to boosting. To test this hypothesis, we evaluated in parallel the post-proliferative tail of the primary response in spleen, LNs, BM and blood, and the kinetics of responsiveness to boost. We identified the time-shift from low to high responsiveness, and characterized the molecular profile of highly responsive splenic memory CD8 T cells.

## Methods

### Adenoviral and MVA vectors

Replication defective, ΔE1 ΔE2 ΔE3 ChAd3 vector encoding HIV-1 gag protein under HCMV promoter (ChAd-gag) and MVA encoding the HIV-1 gag protein under the control of vaccinia p7.5 promoter (MVA-gag) were generated as described and used in all experiments (14–17).

### Vaccination

Six-week-old female BALB/c mice were purchased from Envigo (S. Pietro al Natisone, Udine, Italy), housed at Plaisant animal facility (Castel Romano, Rome, Italy), and used for experiments at 7-9 weeks of age. Mice were divided into groups of at least 35 mice each (untreated and vaccinated). All mice of the vaccinated group were primed at day (d) 0 with ChAd-gag, and either analyzed at the indicated times post-prime, i.e. d30 (range 27-35), d60 (range 60-67), or d100 (range 95-109), or boosted. For prime/boost experiments, primed mice were divided in different sets, each boosted at a single time after prime, i.e. at either d30 or d100 (ranges as above). Viral vectors were administered intramuscularly (i.m.) in the quadriceps at a dose of 10^7^ viral particles (vp) for ChAd-gag and 10^6^ plaque-forming units (pfu) for MVA-gag, in a volume of 50 μl per side (100 μl total) (14). All experimental procedures were approved by the local animal ethics council and performed in accordance with national and international laws and policies (UE Directive 2010/63/UE; Italian Legislative Decree 26/2014; authorization n. 1065/2015-PR).

### Organs

Spleen, LNs, BM and blood were analyzed either at the indicated days after prime or at d45 (range 41-46) after boost. At each time, organs were collected from 3 vaccinated and 3 untreated mice, and cells from the 3 mice of each group were pooled. Spleen, LNs (iliac and inguinal), and blood were processed as described (14, 18). Femurs and tibias were cleared of muscle tissues, and cut at the extremities. The open bone was placed in a cut pipette tip, placed in a microfuge tube, thereby keeping the bone away from the bottom of the tube and allowing the BM to be centrifuged out of the bone at 800x*g* for 1 minute. The bone was discarded and the pellet resuspended, thus obtaining a single cell suspension of BM cells (19). All cell suspensions were prepared in RPMI medium with 2 mM L-Glutamine, 100 U/ml Penicillin, 100 µg/ml streptomycin, 50 µM β-Mercaptoethanol + 10% volume/volume (v/v) Fetal Bovine Serum (FBS), and filtered with pre-separation filters (70 µm) (Miltenyi Biotech, Bergisch Gladbach, Germany).

### Cell membrane and Ki-67/DNA staining

Cells were incubated with purified anti-mouse CD16/CD32 clone 2.4G2 (Fc block; BD Biosciences, San Jose, CA, USA), and stained as described with H-2k(d) AMQMLKETI (gag_197-205_) allophycocyanin (APC)-labeled Tetramer (Tetr-gag, NIH Tetramer Core Facility, Atlanta, GA, USA) and phycoerythrin (PE)-labeled Pentamer (Pent-gag, Proimmune, Oxford, UK), fluorochrome conjugated monoclonal Antibodies (mAbs) against surface (CD3, CD8, CD127, CD62L) and intracellular (Ki-67) molecules, and Hoechst 33342 (Thermo Fisher Scientific, Waltham, MA, USA) (14). The following mAbs were used: anti-CD3ε peridinin chlorophyll protein (PerCP)-Cy5.5 (clone 145-2C11, BD Biosciences), anti-CD8α BUV805 (clone 53-6.7, BD Biosciences), anti-CD127 biotin (clone A7R34, eBioscience, Thermo Fisher Scientific) plus Streptavidin PE-Cy7 (BD Biosciences), or anti-CD62L PE-Cy7 (clone MEL-14, Biolegend, San Diego, CA, USA), and anti-Ki-67 mAb conjugated with Fluorescein isothiocyanate (FITC) or Alexafluor 700 (clone SolA-15; eBioscience, Thermo Fisher Scientific). Dead cells were excluded with eBioscience Fixable Viability Dye eFluor780 (eFluor780, Invitrogen, Thermo Fisher Scientific)

### Intracellular IFN-γ assay

Spleen and BM cells were incubated at 37°C in 5% CO_2_ in round-bottom 96-well plates (2×10^6^ cells/well) for 5 hours with a pool of gag protein-derived peptides (gag peptide pool, 15mers overlapping by 11 amino acids) at final concentration of 2 µg/ml for each peptide. Dimethyl sulfoxide (DMSO, Sigma-Aldrich, St. Louis, MO, USA), the peptide pool diluent, was used as negative control and phorbol myristate acetate/ionomycin (PMA/Iono, Sigma-Aldrich) at final concentration of 20 ng/ml and 1 µg/ml respectively as positive controls. All incubations were performed in the presence of Golgi plug (BD Biosciences). After stimulation, cells were collected and incubated with Fc block, stained with Live/Dead Fixable Violet Dye (Invitrogen, Thermo Fisher Scientific) for viability, and with the following mAbs against surface markers: anti-CD3ε APC, clone 145-2C11; anti-CD8α PerCP, clone 53-6.7; anti-CD4 PE, clone H129.19 (all from BD Biosciences). Intracellular staining was performed after treatment with Cytofix/Cytoperm and in the presence of PermWash (BD Biosciences) using anti-mouse IFN-γ FITC, clone XMG1.2 (BD Biosciences).

### *In vivo* killing

Female BALB/c mice were primed with ChAd-gag and boosted with MVA-gag as above. At d45 (range 43-50) post-boost, vaccinated mice and control untreated mice were injected intravenously (i.v.) with 20x10^6 spleen cells previously obtained from untreated female BALB/c mice and stained with Carboxyfluorescein succinimidyl ester (CFSE, eBioscience, Thermo Fisher Scientific). In more details, the injected cells consisted in a 1:1 mixture of CFSE^high^ gag-peptide pulsed and CFSE^low^ unpulsed cells. Spleen, LNs and BM cells were obtained 3 hours after injection and analyzed by flow cytometry using a Beckman Coulter Cytoflex instrument. Propidium Iodide (PI) was used for dead cell exclusion. Percentage of gag-specific killing was calculated according to (20).

### Flow cytometry analysis

Samples were analyzed by either LSRFortessa flow cytometer (BD Biosciences) or CytoFLEX System B5-R3-V5 (Beckman Coulter, Brea, CA, USA). In some experiments, CD3^—^ cells were gated out when acquiring spleen and BM samples. Data were analysed using FlowJo software, v. 10.7.1 and 10.8.1 (FlowJo, Ashland, OR, USA).

### Estimates of absolute cell numbers

Cells from spleen, LNs and BM were counted by trypan blue exclusion under light microscope, after lysis of Red Blood Cells (RBCs) (Sigma-Aldrich). Residual RBCs —identified as Ter119^+^ CD45^—^ cells by staining with anti-CD45 Alexafluor 488 (clone 30-F11, Biolegend) and anti-Ter119 PerCP-Cy5.5 (clone TER119, eBioscience), and flow cytometry analysis— were found after RBC lysis in spleen (on average 40%) and BM (on average 30%), but not in LNs. Thus, spleen and BM cell counts were multiplied by 0.6 and 0.7, respectively, to obtain nucleated cell counts. Mouse White Blood Cell (WBC) counts/µl and total blood volume were previously reported (21, 22). The absolute numbers of gag-specific CD8 T cells in spleen, LNs, BM and blood, and of IFN-γ^+^ CD8 T cells in spleen and BM, were estimated based on their percentages determined by flow cytometry and on nucleated cell counts of corresponding organ as previously described (23).

### Cell sorting, RNA sequencing, and bioinformatic analysis

Female BALB/c mice were primed with ChAd-gag as above, and analyzed at either d30 (range 28-29) or d100 (range 96-107) post-prime. CD8 T cells were enriched from pooled RBC-lysed spleen cells of 12 primed mice by negative selection with mouse CD8 Dyna Beads magnetic beads (Thermo Fisher Scientific). Enriched CD8 T cells were stained with Tetr-gag APC, Pent-gag PE, anti-CD3ε PerCP-Cy5.5, anti-CD8α FITC mAbs, and eFluor780. Then live Tetr-gag^+^Pent-gag^+^CD3^+^CD8^+^ cells were sorted by flow cytometry into PBS buffer 1% BSA 2 mM EDTA using a FACSAria III (BD Biosciences) equipped with 488, 561, and 633 nm lasers, and with FACSDiva software (BD Biosciences, v6.1.3). To reduce stress, cells were sorted in gentle FACS-sorting conditions, using a ceramic nozzle of size 100 μm, a low sheet pressure of 19.84 pound-force per square inch (psi) that keeps the sample pressure at 18.96 psi and an acquisition rate of maximum 1500 events/sec. FACS-sorted cells were confirmed to be 92.92 ± 5.70% pure prior to RNA extraction. Cells were centrifuged for 5 minutes at 300x*g* and pellets resuspended in Buffer RLT Plus (RNeasy Plus Mini Kit from Qiagen, Germantown, MD, USA), and frozen at -80°C. Total RNA was isolated, and cDNA libraries prepared using NEBNext Single Cell/Low Input Library Prep Kit (from 2ng RNA normalised input), following manufacturer’s instructions. They were then sequenced on Illumina HiSeq 4000 with 100bp single-end reads, following manufacturer’s instructions. Read adaptor removal and quality trimming was carried out with Trimmomatic (version 0.36) (24). Reads were then aligned to the mouse genome, using Ensembl GRCm38 - release 95 as reference. Read alignment and gene level quantification was performed by STAR alignment (v.2.5.2a) (25) together with RSEM package (v.1.2.31) (26). Statistical analyses were performed in the R programming environment (v. 4.0.3). We used DESeq2 (v.1.30.1) to find differential genes using the negative binomial distribution to model counts, with IHW (v.1.18.0) to control the false discovery rate (FDR), and ashr (v. 2.2.47) for effect-size shrinkage. Genes were designated as differentially expressed (DEGs) if FDR ≤ 0.01. All log-fold-changes estimates were regularised using ashr. We used an independent filter of low-signal genes whose effect varied from comparison to comparison, thus the total number of genes was not the same when analyzing down-regulated and up-regulated genes (total number of genes 10,333 and 7,846, respectively) (RNAseq data are available at GEO, access number GSE207389)

### Statistical analysis

Student t test was used for comparison between two groups whenever each group had ≥9 samples, after checking that distribution was normal by Shapiro-Wilk test. Non-parametric tests were used for the remaining comparisons. Mann-Whitney test or Wilcoxon test were used for comparison between two groups. Either Kruskal-Wallis or Friedman test with Dunn’s correction for multiple comparison were used for comparison among more than two groups. Differences were considered significant when * *P* ≤ 0.05; ** *P* ≤ 0.01. Statistical analysis was performed using Prism v.6.0f, GraphPad Software (La Jolla, CA, USA).

## Results

### Kinetics of gag-specific CD8 T cell expansion and re-entry in a resting state after prime

**Figure 1:**
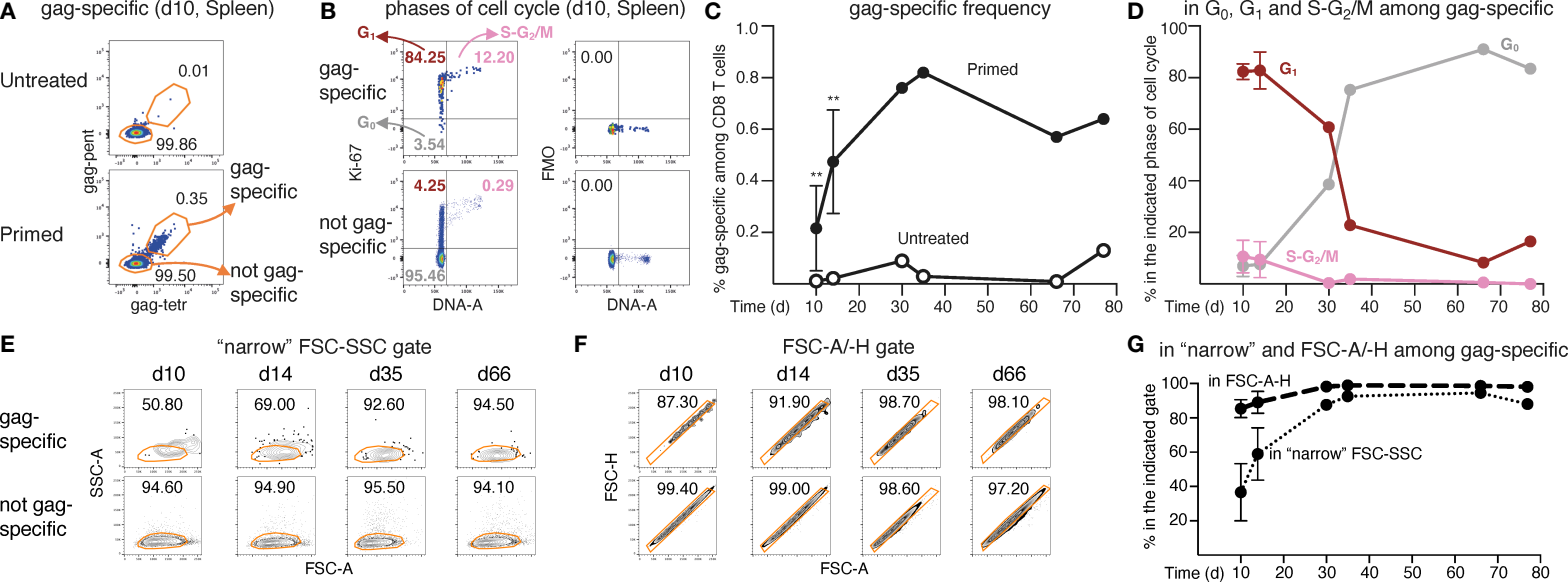
Analysis of frequency and cell cycle of gag-specific CD8 T cells from ChAd-gag-primed mice. Female BALB/c mice were primed i.m. in the quadriceps with ChAd-gag (10^7^ vp) at d0 and the kinetics of gag-specific CD8 T cell response was tracked by flow cytometry. **(A–G)**. Spleen cells were analysed by membrane and Ki-67/DNA staining at different days (d) post-prime (gating strategy in **Figure S1A**). Typical plots showing the percentage of “gag-specific” and “not gag-specific cells” from untreated (top) and primed (bottom) mice at d10 **(A)**. gag-specific cells from primed mice were further analyzed on DNA/Ki-67 plots as follows (**B**, top left panel): cells in the G0 phase of cell cycle were identified as DNA 2n/ Ki-67^—^ (bottom left quadrant); cells in G1 as DNA 2n/ Ki-67^+^ (upper left quadrant); cells in S-G2/M as DNA>2n/ Ki-67^+^ (top right quadrant). As a comparison, not gag-specific cells (**B**, bottom left panel) are shown. For each DNA/Ki-67 plot, corresponding Ki-67 Fluorescence Minus One (FMO) control plots (**B**, right panels) are shown. Summary of the kinetics of gag-specific frequency in primed and untreated mice **(C)**, and of cell cycle phases of gag-specific CD8 T cells in primed mice **(D)** (summary of cell cycle phases of not gag-specific CD8 T cells shown in **Figure S1B**). Kinetics of the percentages of gag-specific and not gag-specific CD8 T cells in the “narrow” FSC-SSC gate (examples in **E**) and in the FSC-A/-H gate (examples in **F**), and summary of gag-specific CD8 T cell results **(G)** (summary of not gag-specific CD8 T cell results shown in **Figure S1E**). The figure summarizes results of 6 independent prime experiments with a total of 84 mice analyzed at the indicated d post-prime. In flow cytometry plots (in **A, B, E, F**), numbers represent percentages of cells in the indicated regions. In panels **(C, D, G)**, symbols at d10 and d14 represent the mean and bars the Standard Deviations of 5 experiments (each performed with pooled cells from 3 mice per group). At d30, d35, d66 and d77, each symbol represents a pool of 3 mice. Statistical analysis was performed using Mann-Whitney test for comparison between untreated and primed mice at d10 and d14 **(C)**, and Wilcoxon test for comparison between gag-specific CD8 T cells in the “narrow” FSC-SSC gate and in the FSC-A/-H gate at d10 and d14 **(G)**. Statistically significant differences are indicated (** P ≤ 0.01). This figure includes unpublished data in relation to (14).

We exploited our recently developed DNA/Ki-67 flow cytometry assay (14, 27, 28) to evaluate the kinetics of response of CD8 T cells specific for the immunodominant gag_197-205_ peptide (gag-specific) in spleen and LNs of BALB/c mice primed with ChAd-gag at about 2 months of age (**Figures 1**, **S1**). As depicted in **Figure S1A**, our strategy of analysis included a DNA-based singlet gate (Step 1), and an unusually “relaxed” FSC-A/SSC-A (FSC-SSC) gate (Step 4, in orange), as opposed to a classical “narrow” gate (Step 4, shown in white for comparison) (14, 27–29). Our strategy was designed to fully detect proliferating cells, following our previous demonstration that T lymphocytes undergoing clonal expansion were only partially included in the commonly used FSC-SSC gates for resting lymphocytes (14, 27–29). Indeed, we previously showed that proliferating lymphocytes had gradually increased FSC and SSC as they progressed into cell cycle, reflecting augmented cell and nucleus size, and mitochondrial dynamics (27).

We then exploited cell staining with MHC-gag multimers to identify gag-specific and not gag-specific CD8 T cells from untreated and primed mice (**Figures 1A**, **S1**, Step 7). Typical examples of DNA/Ki-67 plots of primed spleens are shown in **Figure 1B**, with gating of gag-specific cells in G_0_, G_1_ and S-G_2_/M phases of cell cycle (top left); not gag-specific cells are shown for comparison (bottom left). Specificity of Ki-67 staining was demonstrated by corresponding Fluorescence Minus One (FMO) plots (**Figure 1B**, right). The frequency of gag-specific CD8 T cells in primed mice spleens increased from d10 to d30, and then was maintained at a plateau of ~0.7% up to ~ 3 months after prime, with a background of 0.0-0.1% in the spleens of age-matched untreated control mice (**Figure 1C**). Early after priming 83% and 10% of spleen gag-specific cells were on average in G_1_ and in S-G_2_/M respectively, with very similar results at d10 and d14. At d30 and later time points, the percentage of cells in S-G_2_/M dropped to virtually none, while that of cells in G_1_ slowly declined. The G_1_ trend was mirrored by the gradual increase of cells in G_0_, which represented the majority of gag-specific cells from day 35 onwards (**Figure 1D**). There was no parallel expansion of not gag-specific cells; in fact, ~93% of these cells was in G_0_, ~7% in G_1,_ and ~0.3% in S-G_2_/M at all time points (**Figure S1B**). We also noticed that changes in membrane expression of two markers of long-term memory, i.e. CD127 (the α chain of IL-7 receptor), and CD62L (a LN homing molecule), tended to be more pronounced in gag-specific spleen cells that were already in G_0_ at d30 post-prime, as compared to those that were in G_1_ (**Figures S1C, D**). CD62L results were particularly intriguing, as it is known that this marker is normally high in naïve CD8 T cells, it is down-modulated upon priming, and then re-expressed by memory cells with a T_CM_ phenotype (30, 31), but there is little information about its correlation with the quiescence phase G_0_. We thus examined it in more details, as explained in the next paragraphs.

To track the kinetics of proliferation followed by re-entry of spleen gag-specific CD8 T cells into a resting state, we exploited FSC and SSC changes associated with cell cycle (14, 27). As mentioned above, proliferating lymphocytes have FSC and SSC features that place many of them out of the conventional FSC- and SSC-based gates (14, 27). Thus, as a guide to evaluate the kinetics of primary response, we examined the exit and gradual re-entry of proliferating cells into two gates commonly used for resting lymphocytes, i.e., either the “narrow” FSC-SSC (**Figure 1E**) or the FSC-A/-H gate (**Figure 1F**). In fact, while these gates are normally applied to total spleen cells for lymphocyte and single cell gating, respectively, we exploited them here to follow Ag-primed T cell changes over time. Only a fraction of spleen gag-specific CD8 T cells was captured by any of these two gates in the second week post-prime (**Figures 1E–G**), as expected (14). This inadequacy seemed more evident in the case of the “narrow” FSC-SSC gate than in that of the FSC-A/-H gate (**Figures 1E–G**). One month after priming (d30-d35), the gag-specific cell percentage within the “narrow” FSC-SSC gate raised to ~88-93%, and that within the FSC-A/-H gate to ~98-99%, thus resembling the corresponding percentage of not gag-specific cells; there was no further change at d66 and d77 (**Figures 1E–G**). This kinetics was not observed in the case of not gag-specific cells that we examined in parallel as a control (**Figure S1E**). Results were similar in draining LNs (**Figures S2A–D**). Further analysis demonstrated that only a small percentage of cells in S-G_2_/M was captured by the FSC-A/-H gate, and almost none by the “narrow” FSC-SSC gate in both LNs (**Figure S2E**) and spleen (**Figure S2F**), in agreement with our previous data (14).

### Kinetics of gag-specific T_CM_-phenotype changes at d30, d60 and d100 post-prime

**Figure 2:**
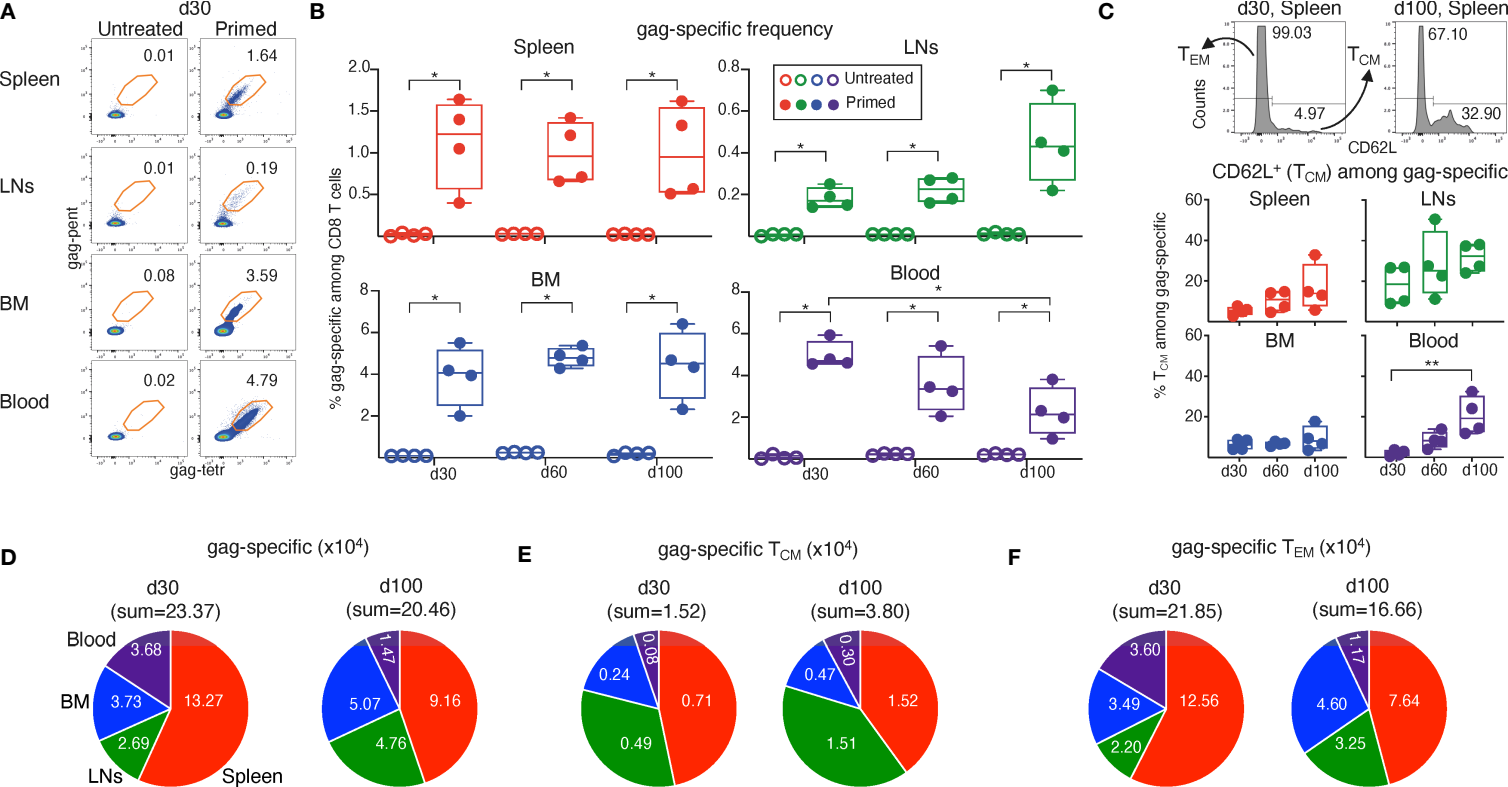
Analysis of T_CM_/T_EM_-phenotype of gag-specific CD8 T cells from ChAd-gag-primed mice, and estimation of absolute cell numbers. Spleen, lymph nodes (LNs), bone marrow (BM) and blood cells from mice primed as in **Figure 1** were analyzed at d30, d60 and d100 post-prime. **(A–C)**. Flow cytometric analysis was performed after membrane and Ki-67 staining (gating strategy in Figure S3, Ki-67 results in Figure 3). Typical plots showing the percentage of gag-specific CD8 T cells at d30 in untreated (left) and primed (right) mice **(A)** and summary of results at d30, d60 and d100 **(B)**. Examples of CD62L histograms of spleen gag-specific CD8 T cells at d30 and d100, showing the gate used to discriminate between TCM (CD62L^+^) and TEM (CD62L^—^) cells (**C**, top). Summary of TCM percentages at d30, d60 and d100 (**C**, bottom). **(D–F)**. Absolute numbers of gag-specific CD8 T cells **(D)**, and of TCM **(E)** and TEM **(F)** gag-specific CD8 T cells, at d30 and d100 in spleen, LNs, BM and blood. The figure summarizes results of 4 independent prime experiments with a total of 72 mice analyzed at the indicated d post-prime. In flow cytometry plots (in **A** and **C**, top), numbers represent percentages of cells in the indicated regions. In panels **(B, C)**, bottom, each symbol represents a pool of 3 mice. Statistical analysis was performed using Mann-Whitney test for comparison between untreated and primed mice **(B)**, and Kruskal-Wallis test with Dunn’s correction for multiple comparison for comparison among primed mice at d30, d60 and d100 (**B, C,** bottom). Statistically significant differences are indicated (* P ≤ 0.05; ** P ≤ 0.01).

We then used flow cytometry to track at d30, d60 and d100 post-prime the frequency of gag-specific cells in spleen, LNs, BM and blood, and the proportions among them of CD62L^+^ cells, i.e. those having a T_CM_ phenotype (30, 31). CD62L staining was also combined with Ki-67 expression analysis, as explained in the next paragraph. It should be noted that in these experiments we did not stain DNA and relied on the typical FSC-A/-H gate to exclude cell aggregates (**Figure S3**, step 1), since we had observed that gag-specific cells in S-G_2_/M were extremely rare after d30 (**Figures 1D**, **S2C**) and that 98-99% of gag-specific cells were comprised in the FSC-A/-H gate at d30 and onwards (**Figures 1F, G**, **S2D**). We used a “relaxed” FSC-SSC gate (**Figure S3**, step 4), that was more appropriate than a “narrow” FSC-SSC gate for evaluation of gag-specific cells in G_1_ (see **Figures S2E, F**), even after d30 when G_1_ mostly represented a post-mitotic state (**Figures 1D**, **S2C**). We found that gag-specific frequency at d30 was on average 5.0% in blood, 3.8% in BM, 1.1% in spleen and 0.2% in LNs from primed mice (examples of flow cytometry plots in **Figure 2A**, summary of results in **Figure 2B**). The frequency remained roughly stable at d60 and d100 in the spleen and BM, with a tendency to increase in the LNs. In contrast, a significant decline was observed in the blood from d30 to d100 (**Figure 2B**). At any time point, and in each organ, primed mice had a significantly higher gag-specific frequency than untreated controls, which always displayed a negligible background (**Figures 2A, B**). In the primed mice samples, we discriminated between T_CM_ and T_EM_ gag-specific CD8 T cells according to their CD62L expression (**Figure 2C**). We found that the proportion of T_CM_ tended to be higher in the LNs, and to increase over time in all organs (**Figure 2C**). Notably, there was a significant rise in T_CM_ proportion among gag-specific CD8 T cells in the blood, from ~2% at d30 to ~21% at d100 on average (**Figure 2C**), reflecting the contemporary changes in lymphoid organs.

To better investigate the d30-d100 shift, we estimated the absolute numbers of gag-specific cells, and of T_EM_ and T_CM_ gag-specific cells in spleen, LNs, BM and blood (**Figures 2D–F**). These estimates took into account CD8 T cell abundance in each organ, and the tendency of CD8 T cell percentages to an age-dependent decline in spleen, LNs and blood but not in BM in the time interval of our study (averages: spleen 9.77%; LN 20.85%; blood 9.25%, BM 0.43% at d30, age 11-13 weeks; spleen 7.50%, LNs 15.15%, blood 8.10%, BM 0.50% at d100, age 21-23 weeks). We found that an overall reduction in gag-specific cell numbers coexisted with selective increases of cells belonging to T_CM_ subset and/or found in certain organs (**Figures 2D–F**). Thus, the sum of gag-specific cells in spleen, LNs, BM and blood at d100 was 88% of that at d30 (**Figure 2D**), and that of T_EM_ 76% of that at d30 (**Figure 2F**). In striking contrast, taking the four organs altogether, T_CM_ cells increased 2.5 times from d30 to d100 (**Figure 2E**). With regards to changes in gag-specific cell distribution, it was remarkable that the sum of cells contained in spleen and blood accounted for ~ ¾ of the cells found in the four organs altogether at d30, and for only ~half of them at d100 (**Figure 2D**). In fact, from d30 to d100 the number of gag-specific cells was reduced in blood and spleen, whereas that in LNs and BM increased (**Figure 2D**). Changes of T_EM_ resembled those of gag-specific cells (**Figure 2F**), whereas a profound T_CM_ cell increase was observed in each organ (**Figure 2E**).

### Kinetics of re-entry into the G_0_ phase of gag-specific CD8 T_CM_ and T_EM_ cells at d30, d60 and d100 post-prime

**Figure 3:**
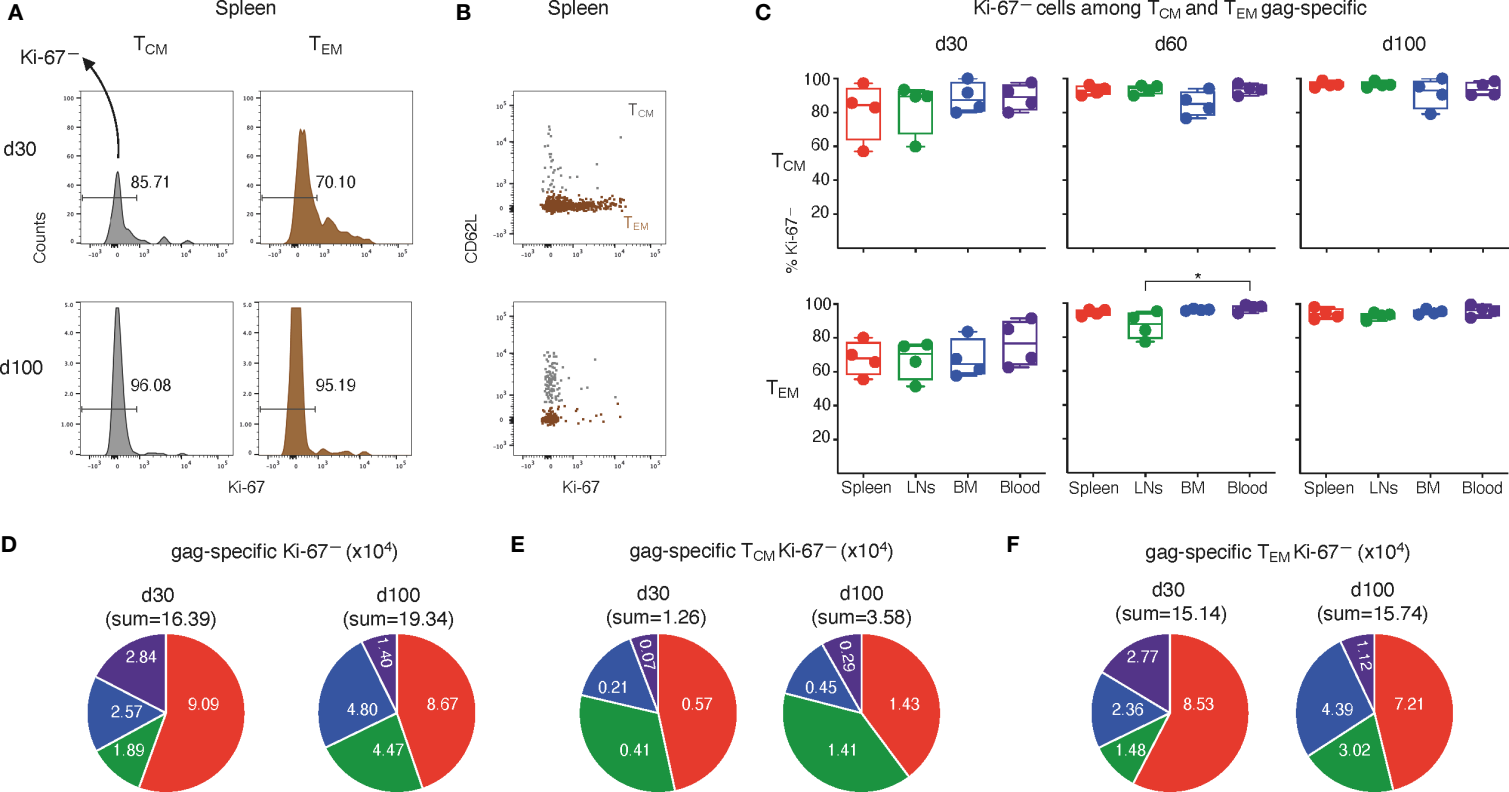
Analysis of Ki-67^—^ cells among T_CM_ and T_EM_-gag-specific CD8 T cells from ChAd-gag-primed mice, and estimation of absolute cell numbers. Spleen, LN, BM and blood cells from primed mice represented in **Figure 2** were analyzed for Ki-67 expression by TCM and TEM gag-specific CD8 T cells, gated as in **Figure 2**. **(A–C)**. Typical histograms showing the percentage of Ki-67^—^ cells among TCM (left) and TEM (right) cells **(A)**, and examples of Ki-67/CD62L plot showing an overlay of TCM (gray) and TEM cells (brown) **(B)**; both panels represent spleen gag-specific CD8 T cells from primed mice at d30 (top) and d100 (bottom). Summary of results of Ki-67^—^ cells among TCM (top) and TEM (bottom) gag-specific CD8 T cells from spleen, LNs, BM and blood at d30, d60 and d100 **(C)**. In **A**, numbers represent percentages of cells in the indicated regions. **(D–F)**. Absolute numbers of gag-specific Ki-67^—^ CD8 T cells **(D)**, and of TCM **(E)** and TEM **(F)** Ki-67^—^ gag-specific CD8 T cells at d30 and d100 in spleen, LNs, BM and blood. Statistical analysis was performed by Friedman test with Dunn’s correction for multiple comparison **(C)**. Statistically significant differences are indicated (* P ≤ 0.05).

We reasoned that the reported higher proliferative response to TCR triggering of T_CM_ cells in comparison with T_EM_ cells (30) might be associated with a diverse kinetics of re-entry into the G_0_ phase after expansion of the two memory cell types. In other words, we hypothesized that a faster re-entry of T_CM_ cells into the quiescent phase G_0_ after priming might be associated with an improved capacity to expand *in vivo* upon antigenic re-exposure, as compared to T_EM_ cells. To identify possible differences in the primary acute response/memory phase transition between the two memory subsets, we tracked Ki-67^—^ (i.e. in G_0_) among T_EM_ and T_CM_ gag-specific cells in the primed mice samples described above. At d30, Ki-67^—^ cells were on average ~81% in T_CM_ and ~68% in T_EM_ gag-specific cells from primed spleen (examples of flow cytometry histograms in **Figure 3A**, top). Both percentages increased over time, being on average ~96% in T_CM_ and ~94% in T_EM_ at d100 (examples in **Figure 3A**, bottom). The marked Ki-67^+^ cell decline among T_EM_ cells from d30 to d100 was concurrent with a higher representation of T_CM_ cells at d100, as evident in a typical Ki-67/CD62L plot showing an overlay of T_CM_ (gray) and T_EM_ (brown) gag-specific cells (**Figure 3B**, see also **Figures 2C–F**). A similar pattern was observed across spleen, LNs, BM and blood, with two points to be highlighted (**Figure 3C**). First, a statistically significant difference between LNs and blood T_EM_ at d60 (bottom center), not observed at d100 (bottom right), indicating a slow re-entry of LN T_EM_ in G_0_. Second, a tendency of BM T_CM_ cells to contain a smaller fraction of Ki-67^—^ than the other organs, both at d60 (top center) and at d100 (top right), indicating a low level of persistent activation of T_CM_ in the BM.

In terms of absolute numbers, the sum of Ki-67^—^ gag-specific cells in spleen, LNs, BM and blood altogether at d100 was 1.2-fold higher than that at d30, as a net result of increase in LNs and BM, almost no change in spleen, and evident reduction in blood (**Figure 3D**). This was in contrast with the absolute number of Ki-67^—^ T_CM_ found in the four organs altogether, that showed a striking 2.9-fold increase from d30 to d100, with a pronounced rise in each organ, especially in blood and LNs (**Figure 3E**). The sum of Ki-67^—^ T_EM_ in the four organs at d100 was similar to that at d30 (**Figure 3F**), in contrast to the above-described reduction of total T_EM_ (**Figure 2F**). Single organ comparison showed that Ki-67^—^ T_EM_ cells were reduced in blood and spleen, but increased in LNs and BM (**Figure 3F**).

### RNA sequencing and bioinformatic analysis of spleen gag-specific CD8 T cells at d30 and d100 after prime

**Figure 4:**
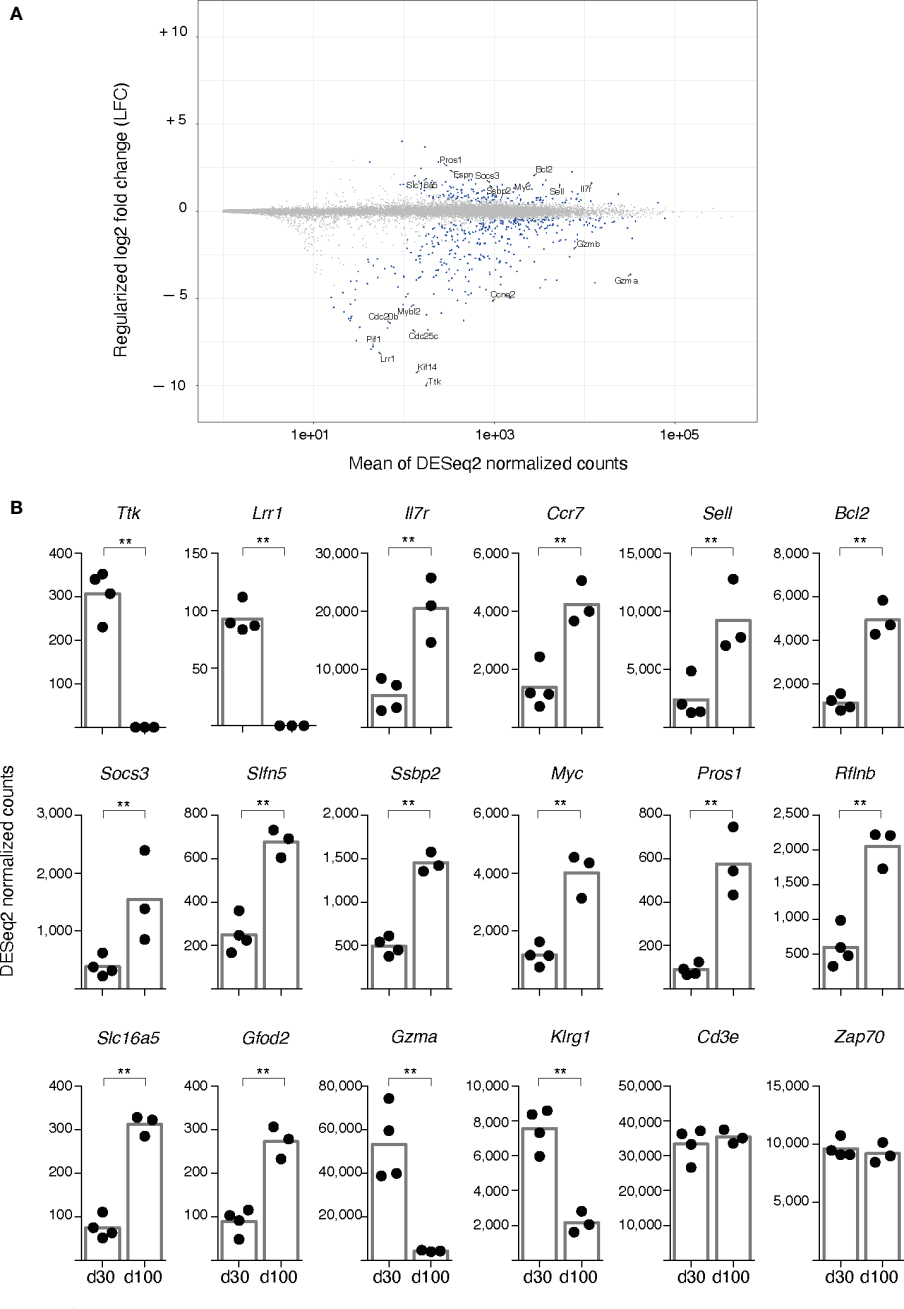
RNA seq analysis of spleen gag-specific CD8 T cells at d30 and d100 post-prime. Bulk RNA was sequenced from sorted spleen gag-specific CD8 T cells at d30 and d100 post-prime, in 4 independent prime experiments with a total of 7 samples. Bioinformatic analysis was performed to compare d100 and d30. **(A)**. Scatterplot of genes whose estimated absolute log-2 fold change was < 11. The y-axis represents Log-2 fold changes regularised using an empirical Bayes method, and the x-axis represents means across all samples of the DESeq2-normalised counts. Differentially expressed genes (DEGs) with a statistically significant difference (false discovery rate (FDR) ≤ 0.01) are highlighted in blue. Selected gene symbols among the top 50 significantly upregulated (top 50-up) and the top 50 significantly downregulated (top 50-down) genes are indicated (see **Figure S4** for full list). **(B)**. DESeq2-normalised counts of representative DEGs having a statistically significant difference between d100 and d30, i.e. Ttk, Lrr1, IL7r, Ccr7, Sell, Bcl2, Socs3, Slfn5, Ssbp2, Myc, Pros1, Rflnb, Slc16a5, Gfod2, Gzma, Klrg1. Analysis of Cd3e and Zap70 is included. Each symbol represents an individual sample; columns represent the mean of d30 and d100 group; statistically significant differences are indicated (** FDR ≤ 0.01).

A transcriptomic comparison between d100 and d30 spleen gag-specific cells by RNAseq showed that samples of the two groups were easily separated by Principal Component Analysis (**Figures 4**, **S4A**). Significant changes at d100 were found in 512 differentially expressed genes (DEGs), 362 of which were down-regulated and 150 up-regulated (**Figure 4A**). The top 50-up and top 50-down DEGs showed comparable results across samples of each group (**Figure S4B**).

A striking reduction was observed in a small set of downregulated DEGs, with some of them showing a regularized log-2 Fold Change (LFC) comprised between -5 and -10 (**Figure 4A**). Among the DEGs that were down to virtually 0 normalized counts, *B-myb* (also known as *Mybl2*) was an already recognized T cell effector player of transition into memory state (32, 33), while others were newly described in this context, e.g. *Ttk*, the gene for Thymidine kinase 1, an IL-2 induced kinase regulating cell cycle progression of T cells (34); *Kif14*, the gene for Kinesin Family Member 14, a positive regulator of cell cycle (35, 36); and *Lrr1*, the gene for Leucine-Rich Repeat Protein 1, an inhibitor of 4-1BB signalling (37) (**Figures 4A, B**). Notably, about half of the top 50 significantly down-regulated genes (top 50-down) encoded for proteins involved in cell proliferation (**Figure S4C**, in bold blue). There was a trend of down-regulation of *Mki67*, the gene coding for Ki-67 protein, that did not reach statistical significance (not shown), even though the percentage of Ki-67^+^ cells within spleen gag-specific CD8 T cells significantly dropped from 31.50 ± 9.16 at d30 to 5.36 ± 2.56 at d100 (averages ± Standard Deviation, Mann-Whitney test, *P* ≤ 0.05). The lack of statistical significance in transcriptomic analysis possibly reflected the correction for multiple tests used for RNAseq data statistical analysis.

In contrast to the dominance of proliferative genes in the top 50-down, the top 50 up-regulated DEGs (top 50-up) were more heterogeneous, and the LFC did not exceed +5 for any of them (**Figures 4A**, **S4C**). As expected, the top 50-up DEGs comprised *Ccr7*, *Sell*, and *Il7r* that encode for CCR7, CD62L, and IL-7Rα (CD127) respectively, all recognized markers for T_CM_ phenotype and memory T cell longevity (33, 38), as well as *Bcl2*, a pro-survival gene (33, 39), and *Socs3*, which encodes for a cytokine signalling regulator that controls IL-7Rα re-expression after initial down-regulation in activated T cells (40) (**Figures 4A, B**). Remarkably, top 50-up DEGs included *Slfn5*, a member of the Schlafen family of genes that has been implicated in T cell quiescence and proliferative potential (41–43), and some genes previously involved in regulating quiescence and maintenance of hematopoietic stem cells (HSCs), i.e. *Ssbp2*, Sequence-specific ssDNA–binding protein 2 (44), and *Myc* (45), often cited for its role in T cell metabolism and memory T cell differentiation (33, 46, 47) (**Figures 4A, B**). The above mentioned *Socs3* has also been implicated in re-setting quiescence of HSCs after proliferation (48), while the pleiotropic gene *Pros1*, Protein S, also in the top 50-up genes, has been proposed as a regulator of neural stem cell equilibrium between quiescence and proliferation (49)(**Figures 4A, B**). Altogether, changes in these genes (**Figures 4A, B**, **S4C**, in bold red) support the notion that the quiescent state of gag-specific cells at d100 was actively and finely regulated.

Additional top 50-up DEGs encoded for proteins regulating cell metabolism and redox state (i.e. *Qpct*, *Slc16a5*, *Gfod2*, *Nmnat3*) (**Figures 4A, B**, **S4C**, in bold black), pointing to a metabolic change at d100. It is worth noting that *Rflnb*, Refilin B, a TGF-β effector (50) and *Tgfbr3*, TGF-β receptor III, were among the top 50-up DEGs, in agreement with the major role of TGF-β signalling in T cell memory (51), and of TGF-β RI and RII in T cell biology (52, 53), even though the function of TGF-β RIII has been poorly investigated in this context (54). Furthermore, *Cd101* was among the top 50-up DEGs; this gene encodes for a T cell-inhibitory glycoprotein shown to come up in chronic infection (55) (**Figures 4B**, **S4B, C**).

Although not listed in the top 50-down, *Gzma*, *Gzmb*, and *Gzmk* were significantly down-regulated, as were *Nkg7* and *Klrg1*, all typical genes of CD8 T cell effector signature (**Figures 4A, B**). Control genes with no significant changes included *CD3d*, *CD3g*, *CD3e*, *ZAP70*, *Cd8a* and *Cd8b1* (**Figure 4B**; RNAseq data available at GSE207389).

### Kinetics of responsiveness to boost

**Figure 5:**
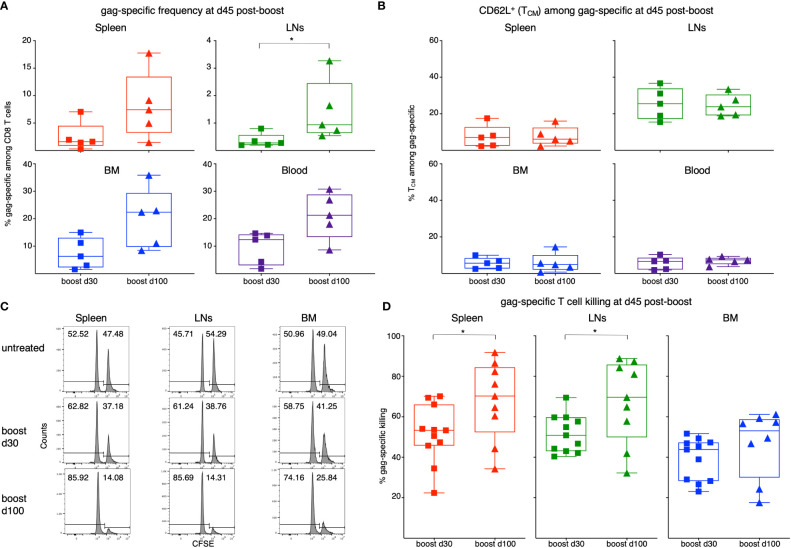
Analysis of gag-specific CD8 T cell frequency, T_CM_-phenotype and *in vivo* killing activity at d45 post-boost. Female BALB/c mice were primed as in **Figure 1** at d0. One set of primed mice was boosted with MVA-gag at d30 post-prime, and another at d100 post-prime. For each set, analysis was performed at d45 post-boost. **(A, B)**. Frequency of gag-specific CD8 T cells **(A)** and percentage of TCM among gag-specific CD8 T cells **(B)** in spleen, LNs, BM and blood of primed/boosted mice. **(C, D)**. Primed/boosted and untreated control mice were injected i.v. with a 1:1 mixture of gag-pulsed CFSE^high^ cells and unpulsed CFSE^low^ syngeneic spleen cells (approximately 10x10^6 cells each). After 3 hours, the percentages of CFSE^high^ and CFSE^low^ cells were measured in spleen, LNs and BM, and the percentage of gag-specific killing was determined. Examples of CFSE histograms **(C)** and summary of results **(D)**. **(A, B)** summarize results of 5 independent prime/boost experiments with a total of 60 mice, including control untreated mice (note that at each time point, 3 untreated mice were examined as a control; results were similar to those of untreated control mice shown in **Figures 2A, B**). Each symbol represents a pool of 3 mice. **(C, D)** summarize results of 4 independent prime/boost experiments with a total of 36 mice, including control untreated mice (see example in panel **C**). In **(C)**, numbers represent percentages of cells in the indicated regions. In **(D)**, each symbol represents a single mouse. Statistical analysis was performed by either Student t test, after checking that distribution was normal by Shapiro-Wilk test, or Mann-Whitney test. Statistically significant differences are indicated (* P ≤ 0.05).

To compare protocols with different prime/boost intervals, ChAd-gag-primed mice were boosted with MVA-gag either at d30 or at d100 post-prime, and rested for 45d. Then the frequency of gag-specific cells, and that of T_CM_ among gag-specific cells, was measured in spleen, LNs, BM and blood (**Figures 5A, B**). There was a trend of higher gag-specific frequency when boost was performed at d100 post-prime as compared to boost at d30 in all organs, that reached statistical significance in LNs (**Figure 5A**). Independently of the time of boost, the organs with the highest frequencies were BM and blood, followed by spleen and then LNs, similarly to the results at d30 and d60 post-prime (see **Figure 2B**). There was no difference between the two boosts in terms of proportion of T_CM_ among gag-specific cells (**Figure 5B**). As expected, LNs contained a higher percentage of T_CM_ than any of the other organs, thus resembling post-prime data at d30 and d60 (see **Figure 2C**).

*In vivo* killing assay showed that gag-specific cells were functional in spleen, LNs and BM (**Figures 5C, D**). There was a significantly higher percentage of gag-specific killing in spleen and LNs in the group of mice boosted at d100 post-prime as compared to the group boosted at d30, and a tendency of similarly increased killing in the BM (**Figure 5D**). Spleen and LNs showed a more prominent killing than BM (**Figures 5C, D**), possibly reflecting their higher CD8 T cell percentages (see above). Intracellular IFN-γ assay performed at d45 post-boost by stimulating either spleen or BM cells with a pool of gag protein-derived peptides (gag peptide pool) confirmed that boost at d100 post-prime elicited a significantly stronger CD8 T cell response than boost at d30 (**Figures S5A–C**). Absolute cell number estimation similarly showed that boost at d100 was more effective than boost at d30 (**Figures S5D–F**), and also demonstrated that both boosts yielded tremendously higher numbers of gag-specific CD8 T cells in spleen and BM than those obtained by prime only (compare **Figure S5E** with **Figure 2D**). In respect to BM versus spleen comparison, when percentage of IFN-γ^+^ among CD8 T cells was evaluated, BM response reached higher levels than that in spleen in both d30 and d100 experimental groups (**Figures S5B, C**), echoing the gag-specific frequency results (see **Figure 5A**). In terms of absolute gag-specific cell numbers, spleen contained 2-4-times more cells than BM (**Figure S5D–F**). Estimations based on IFN-γ^+^ assay (**Figures S5D, F**) were somehow different as compared to those based on MHC-gag multimer staining (**Figure S5E**), even though cell numbers were in a similar range. This was not surprising, considering that IFN-γ^+^ assay measured CD8 T cells producing one cytokine after stimulation with gag peptide pool, whereas MHC-gag multimer staining detected CD8 T cells specific for a single immunodominant peptide, i.e., gag_197-205_. Altogether, these results show that boost at d100 was more effective than that at d30.

## Discussion

An improved understanding of memory CD8 T cell biology can be highly beneficial for prevention and treatment of human infections, autoimmune diseases and cancers. Long-lived CD8 T cell memory can be established by vaccination protocols based on at least two vaccine doses, however decisions regarding prime/boost time interval have been taken mostly empirically so far (56, 57). In this article we described a splenic memory CD8 T cell signature associated with enhanced response to delayed boost in a model of ChAd-gag/MVA-gag vaccination of BALB/c mice.

In our model, delayed boost at d100 post-prime was more effective than early boost at d30 in terms of frequency and numbers of gag-specific CD8 T cells, *in vivo* gag-specific killing, and IFN-γ production, all measured at d45 post-boost, i.e. in the secondary memory phase. In contrast, delayed and early boost resulted in similar proportions of T_CM_ among gag-specific CD8 T cells in lymphoid organs, suggesting that a more effective response following boost at d100 did not include a change in T_CM_ representation at d45 post-boost.

In our experiments, mice were primed at ∼2 months of age, and they reached ∼3 and 5 and ½ months when they were boosted at d30 and d100 post-prime, respectively. In agreement with the previously described naïve CD8 T cell decay in aging mice, in relation with reduced thymic output (58, 59), we found here that CD8 T cell percentages tended to decrease at d100 in spleen, LNs and blood, but not in the BM. Nevertheless, considering the limited time frame of our investigation, and that the previously described decay involved naïve cells but not memory CD8 T cells (58), we did not expect any impact of CD8 T cell percentage decrease on gag-specific cell response to boost. As a matter of fact, we found that responsiveness to boost was increased at d100.

We identified a d100 splenic transcriptomic profile of gag-specific memory CD8 T cells, characterized by shut off of several proliferative genes (e.g. *Ccna2*, *Cdc25c*, *Pclaf*), and up-regulation of stem cell genes previously implicated in setting the equilibrium between quiescence and proliferation (e.g. *Slfn5, Ssbp2*, *Myc, Socs3*). These results are in agreement with the absolute dominance of Ki-67^—^ cells by flow cytometry analysis, and give granularity to the molecular changes occurring from d30 to d100, i.e. a time interval remarkably characterized by stable numbers of Ki-67^—^ gag-specific CD8 T cells in the spleen. Transcripts involved in T cell negative regulation and TGF-β pathway (e.g. *Rflnb, Tgfbr3, Cd101*) were also up-regulated, as were some metabolic genes (e.g. *Qpct*, *Gfod2*). Increased expression of T_CM_/long-lived memory T cell markers (e.g. *Sell*, *Ccr7*, *Il7r*, *Bcl2*), and down-regulation of effector T cell transcripts (e.g. *Gzma, Gzmb, Gzmk*, *Nkg7*, *Klrg1*) were in agreement with previous results (33, 60). At first, T_CM_ marker up-regulation at d100 might appear in contrast with the concurrent strong down-regulation of proliferative genes, considering that T_CM_ phenotype has been associated with increased proliferation potential (30). Nonetheless, these apparent puzzling findings might be reconciliated according to a recent hypothesis suggesting that persistence of memory CD8 T cells in a non-proliferative quiescent state might preserve their potential for prompt expansion upon secondary antigen stimulation (61).

Our flow cytometry analysis and our cell number estimates suggest that gag-specific CD8 T cells migrated from spleen and blood into LNs and BM during the d30-d100 time frame, even though migration in and out of other organs cannot be excluded. Our further findings on gag-specific T_CM_/T_EM_ phenotypes are consistent with the possibility that T_CM_ had a survival advantage over T_EM_ in all examined organs, and likely self-renewed in the BM (61), while a fraction of T_EM_ up-regulated CD62L, thus acquiring a T_CM_ phenotype. Thus, the transcriptomic differences between d100 and d30 spleen gag-specific CD8 T cells might be due to: i) plasticity of the splenic memory population (e.g. T_EM_ to T_CM_ shift); ii) selective survival of a cell subset (e.g. Ki-67^—^ T_CM_); iii) selective cell recirculation in and out of spleen (e.g. T_EM_ migration out of the spleen and accumulation into LNs and BM); iv) a combination of some or all of the above.

**Figure 6:**
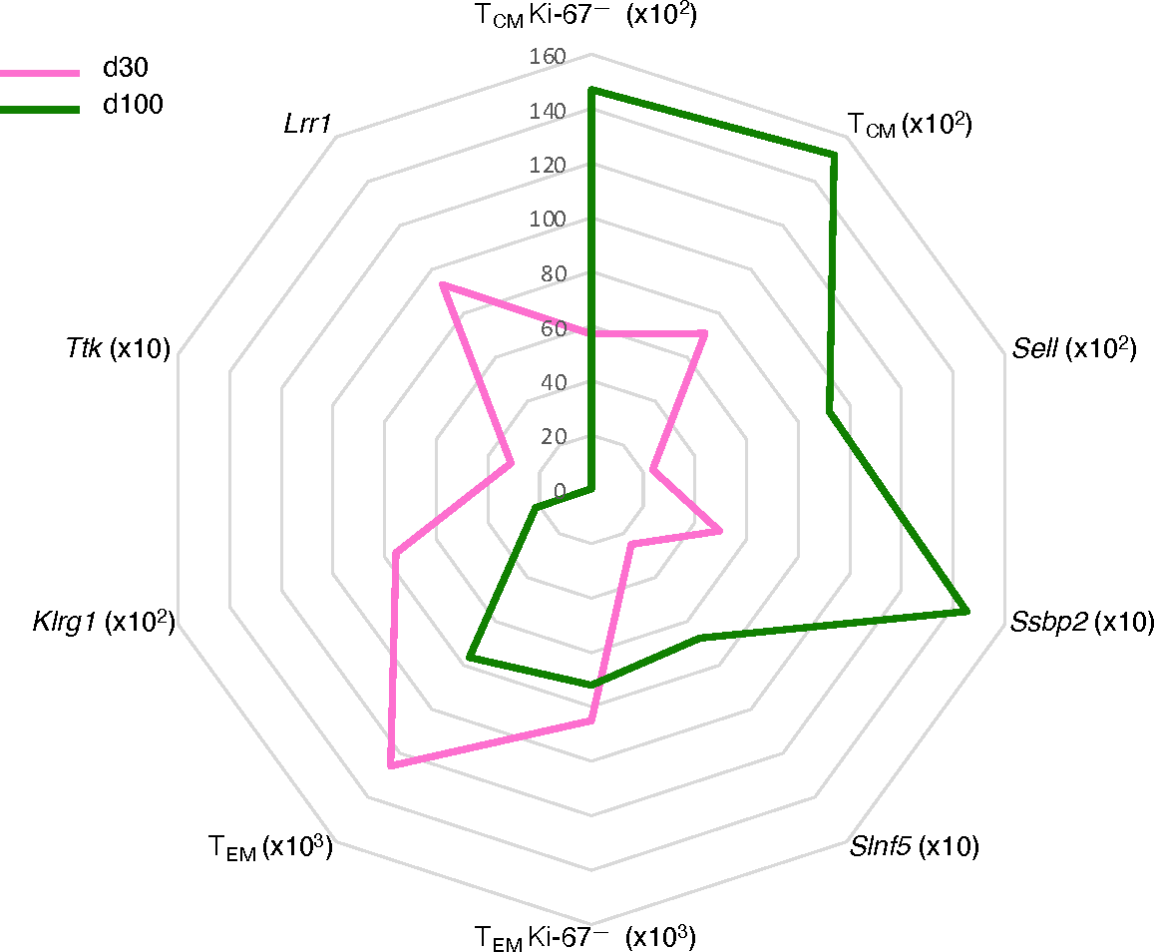
Schematic of “quiescent-but highly responsive” memory spleen CD8 T cell signature. Spider plot representing key features of splenic signature of gag-specific CD8 T cells at d30 (pink) and d100 (green) post-prime. Units of measure are the following: absolute cell numbers in the spleen for T_CM_, T_EM_, Ki-67^—^ TCM, and Ki-67^—^ TEM gag-specific CD8 T cells (see **Figures 2E, F**, **3E, F**); mean DESeq2-normalized counts for Sell, Ssbp2, Slnf5, Klrg1, Ttk, and Lrr1 (see **Figure 4B**).

In sum, the main features of the d100 versus d30 gag-specific CD8 T cells in the spleen were a selective 2.6-fold increase in Ki-67^—^ T_CM_ as opposed to a 1.2-fold decrease in Ki-67^—^ T_EM_, and a transcriptional switch to a mature memory state (**Figure 6**). This included, as expected, an increase in T_CM_ phenotype and a decrease in T cell effector genes, plus newly described changes in genes regulating quiescence and proliferation, not implicated before in T cell memory, and in transcripts involved in inhibitory pathways of T cell responses (**Figure 6**). It is remarkable that in parallel with the d100-d30 molecular shift in the spleen, we measured significant changes in the blood, i.e. a decline in gag-specific frequency within CD8 T cells, and an increase of T_CM_ within gag-specific cells.

Our data are in the same line of previous evidence showing compromised memory T cell longevity with short 14d-prime/boost intervals (62). However, we specifically addressed memory CD8 T cell maturation, as we focused on ≥ 30 d post-prime, after the ending of clonal expansion (63). Some older studies also compared a d30 prime/boost interval with longer ones, using adenoviral vector-based vaccine in mouse models, e.g., d30 versus d60 in ref (9), and ∼d30 versus ∼d110 in ref (8). Some key features characterize our study as compared to these previous ones. Our choice of d30 versus d100 prime/boost comparison was based on the hypothesis that establishment of quiescence in primed CD8 T cells would be related to improved cells’ responsiveness to boosting, and on the experimental results on the kinetics of accumulation of Ki-67^—^ T_CM_ and T_EM_ gag-specific CD8 T cells in lymphoid organs. In contrast, kinetics of clonal expansion and of re-entry in quiescent phase were not investigated in the above-mentioned studies, nor advanced technologies such as RNAseq were used to characterize the molecular signature of highly responsive memory CD8 T cells at d100 post-prime (8, 9). Furthermore, we compared secondary responses at d45 post-boost, in contrast to the above-mentioned comparative studies that analyzed earlier times post-boost (8, 9). This is because we were interested in established CD8 T cell memory, rather than in changes of the kinetics and/or magnitude of the acute response after boost.

Our data are in agreement with old pioneer studies and more recent ones that altogether emphasize through different approaches the role of a T_CM_/stem cell molecular profile for long-lived T cell memory (60, 64–67), and are consistent with the hypothesis that lymphoid microenvironments regulate the equilibrium between quiescence and self-renewal in long-term T cell memory (61). Notably, our findings establish a new memory CD8 T cell profile of responsiveness to boost, giving a valuable contribution to the rational design of vaccination protocols. Advancements in this field are much needed, as defining the time interval between vaccine shots represents one of the current challenges after the success of many anti-SARS-CoV-2 vaccination strategies, including those based on adenoviral-vectors and on mRNA (13).

## Data availability statement

The datasets presented in this study can be found in online repositories. The name of the repository and accession number can be found below: NCBI Gene Expression Omnibus; GSE207389.

## Ethics statement

All experimental procedures were approved by the local animal ethics council and performed in accordance with national and international laws and policies (UE Directive 2010/63/UE; Italian Legislative Decree 26/2014; authorization n. 1065/2015-PR).

## Author contributions

ANat and FD conceived the project, designed experiments, interpreted the results, and wrote the paper with help by SS. AF, SC, RS and ANic provided the viral vectors and conducted/supervised mouse immunizations. ANat, SS, GF, LL, AMC performed/analysed flow cytometry experiments. ANat, GP, MM-R, GK performed/analysed cell sorting and RNAseq experiments. AMC, RS, SB performed/analysed intracellular IFN-γ assay. AF, SC, AS and ACH advised on data discussion and paper writing. ACH advised on concepts to prioritize in data analysis and paper writing. All authors contributed to the article and approved the submitted version.
