# Corrigendum: Improved memory CD8 T cell response to delayed vaccine boost is associated with a distinct molecular signature

**DOI:** 10.3389/fimmu.2023.1199754

**Published:** 2023-05-02

**Authors:** Ambra Natalini, Sonia Simonetti, Gabriele Favaretto, Lorenzo Lucantonio, Giovanna Peruzzi, Miguel Muñoz-Ruiz, Gavin Kelly, Alessandra M. Contino, Roberta Sbrocchi, Simone Battella, Stefania Capone, Antonella Folgori, Alfredo Nicosia, Angela Santoni, Adrian C. Hayday, Francesca Di Rosa

**Affiliations:** ^1^ Institute of Molecular Biology and Pathology, National Research Council of Italy (CNR), Rome, Italy; ^2^ Department of Molecular Medicine, University of Rome “Sapienza”, Rome, Italy; ^3^ Center for Life Nano- & Neuro-Science, Fondazione Istituto Italiano di Tecnologia (IIT), Rome, Italy; ^4^ Immunosurveillance Laboratory, The Francis Crick Institute, London, United Kingdom; ^5^ Bioinformatic and Biostatistics Science and Technology Platform, The Francis Crick Institute, London, United Kingdom; ^6^ ReiThera S.R.L., Rome, Italy; ^7^ CEINGE, Naples, Italy; ^8^ Department of Molecular Medicine and Medical Biotechnology, University of Naples Federico II, Naples, Italy; ^9^ IRCCS Neuromed, Isernia, Italy; ^10^ Peter Gorer Department of Immunobiology, King’s College London, London, United Kingdom; ^11^ National Institute for Health Research (NIHR), Biomedical Research Center (BRC), Guy’s and St Thomas’ NHS Foundation Trust and King’s College London, London, United Kingdom

**Keywords:** CD8 T cells, memory, prime-boost interval, transcriptomic profile, vaccination

In the published article, there was an error in **Figure 5**, panel A as published. The mistake was to show a statistically significant difference in the spleen, as indicated by an asterisk in panel A, top left. The correct panel A doesn’t show a statistically significant difference in the spleen, but only in the LNs, in agreement with the text of the result section, as in the sentence “There was a trend of higher gag-specific frequency when boost was performed at d100 post-prime as compared to boost at d30 in all organs, that reached statistical significance in LNs (**Figure 5A**).” The corrected **Figure 5** and its caption appear below.

**Figure 5:**
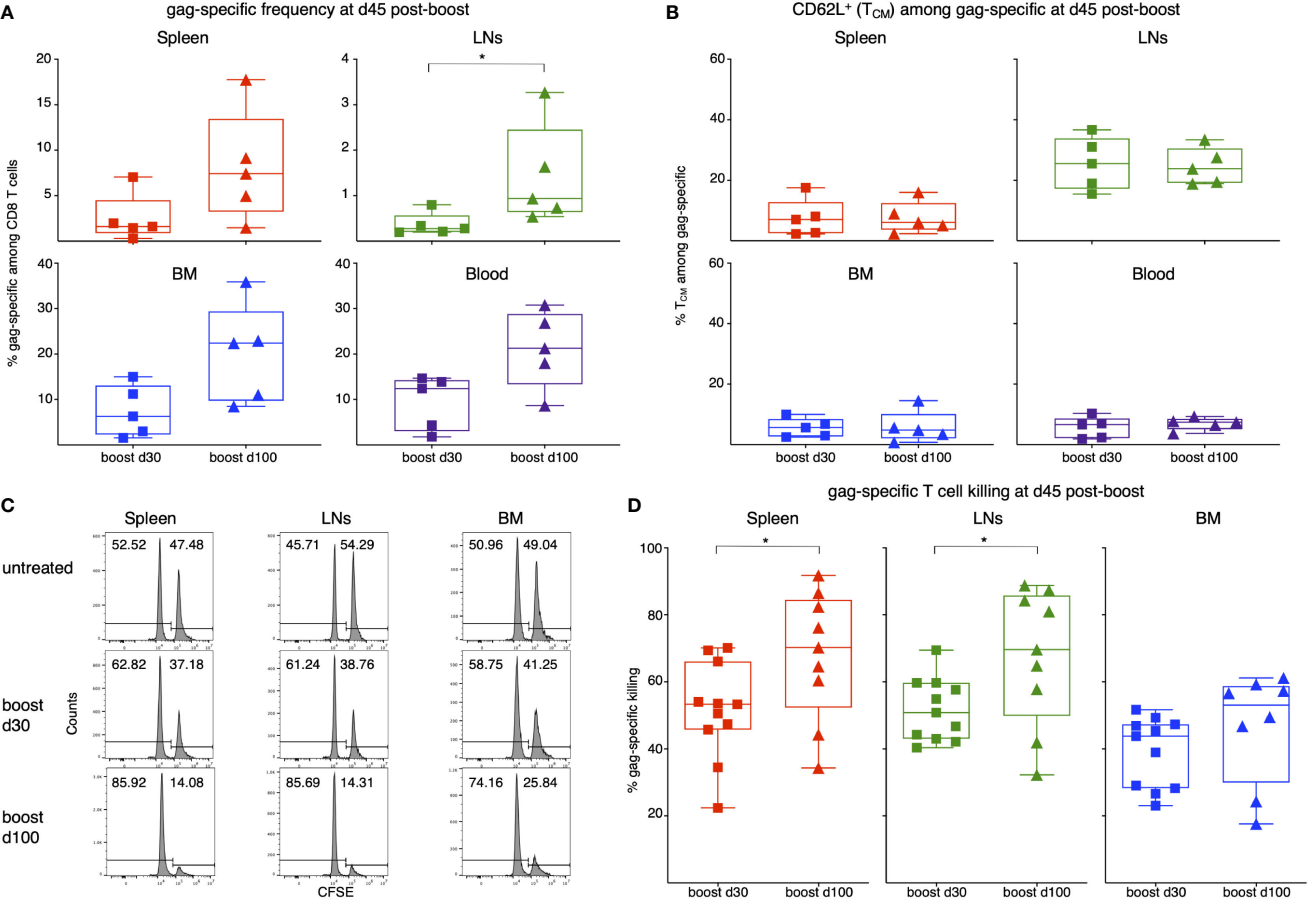
Analysis of gag-specific CD8 T cell frequency, T_CM_-phenotype and *in vivo* killing activity at d45 post-boost. Female BALB/c mice were primed as in **Figure 1** at d0. One set of primed mice was boosted with MVA-gag at d30 post-prime, and another at d100 post-prime. For each set, analysis was performed at d45 post-boost. **(A, B)**. Frequency of gag-specific CD8 T cells **(A)** and percentage of T_CM_ among gag-specific CD8 T cells **(B)** in spleen, LNs, BM and blood of primed/boosted mice. **(C, D)**. Primed/boosted and untreated control mice were injected i.v. with a 1:1 mixture of gag-pulsed CFSE^high^ cells and unpulsed CFSE^low^ syngeneic spleen cells (approximately 10x10^6 cells each). After 3 hours, the percentages of CFSE^high^ and CFSE^low^ cells were measured in spleen, LNs and BM, and the percentage of gag-specific killing was determined. Examples of CFSE histograms **(C)** and summary of results **(D)**. **(A, B)** summarize results of 5 independent prime/boost experiments with a total of 60 mice, including control untreated mice (note that at each time point, 3 untreated mice were examined as a control; results were similar to those of untreated control mice shown in **Figures 2A, B**). Each symbol represents a pool of 3 mice. **(C, D)** summarize results of 4 independent prime/boost experiments with a total of 36 mice, including control untreated mice (see example in panel **C**). In **(C)**, numbers represent percentages of cells in the indicated regions. In **(D)**, each symbol represents a single mouse. Statistical analysis was performed by either Student t test, after checking that distribution was normal by Shapiro-Wilk test, or Mann-Whitney test. Statistically significant differences are indicated (*P ≤ 0.05).

The authors apologize for this error and state that this does not change the scientific conclusions of the article in any way. The original article has been updated.

