# Supplementary material for: Improved memory CD8 T cell response to delayed vaccine boost is associated with a distinct molecular signature
Source: Front Immunol. 2023 Feb 14;14:1043631. doi: 10.3389/fimmu.2023.1043631 (PMC9973452; doi:10.3389/fimmu.2023.1043631)
Supplement: Supplementary file 1 [file DataSheet_1.pdf]

Supplementary material for

Ambra Natalini *et al*
"Improved memory CD8 T cell response to delayed vaccine boost is associated with a distinct molecular signature"
Frontiers in Immunology (2023) doi: 10.3389/fimmu.2023.1043631

- Figure S1
- Figure S2
- Figure S3
- Figure S4
- Figure S5
- Supplementary Figure legends

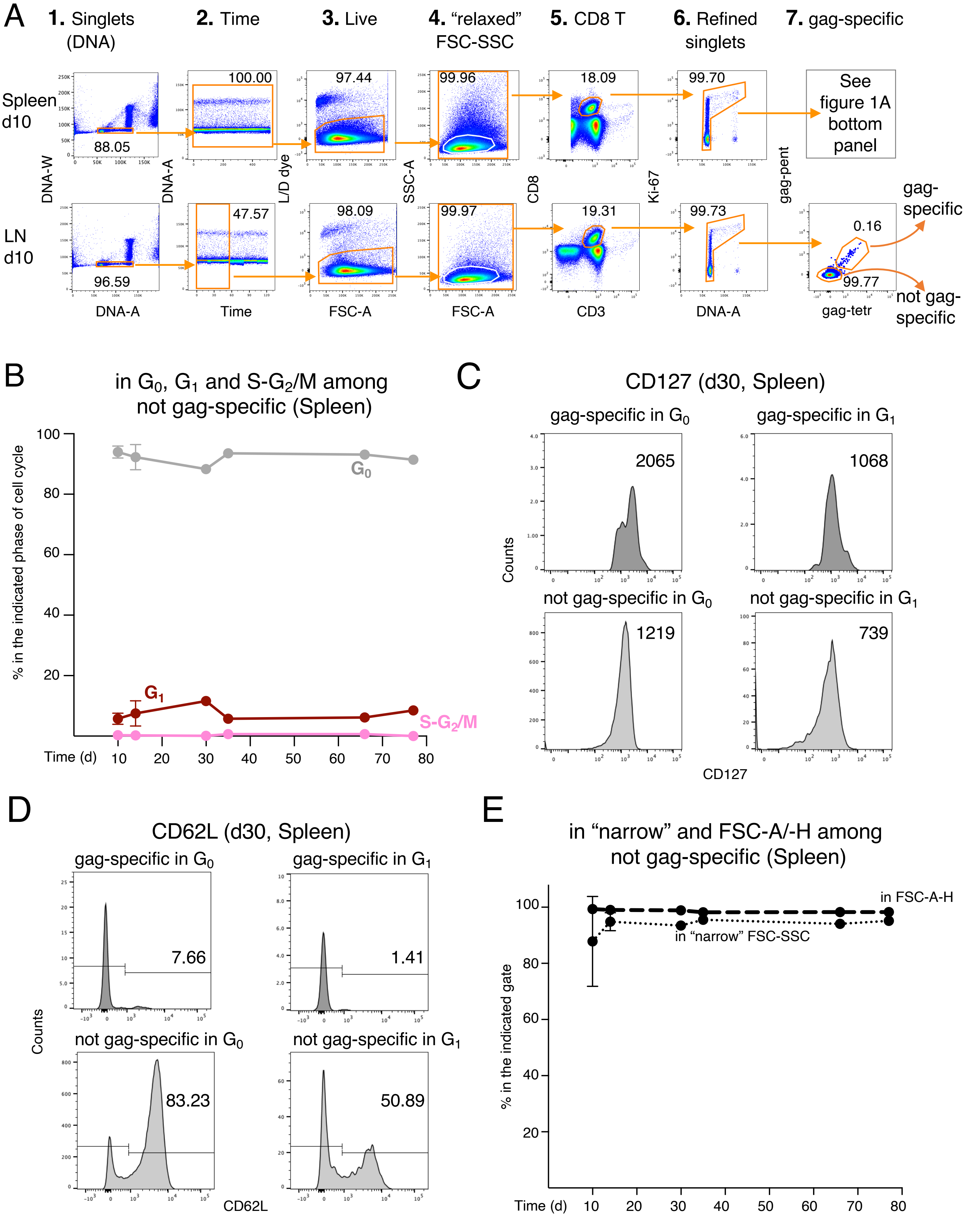
A
1. Singlets (DNA)
2. Time
3. Live
4. "relaxed" FSC-SSC
5. CD8 T
6. Refined singlets
7. gag-specific
Spleen d10
LN d10
88.05
100.00
97.44
99.96
18.09
99.70
See figure 1A bottom panel
96.59
47.57
98.09
99.97
19.31
99.73
0.16
99.77
gag-specific
not gag-specific
DNA-W
DNA-A
L/D dye
SSC-A
CD8
Ki-67
gag-pent
Time
FSC-A
CD3
gag-tetr
B
in G0, G1 and S-G2/M among not gag-specific (Spleen)
% in the indicated phase of cell cycle
G0
G1
S-G2/M
Time (d)
C
CD127 (d30, Spleen)
gag-specific in G0
gag-specific in G1
not gag-specific in G0
not gag-specific in G1
2065
1068
1219
739
Counts
CD127
D
CD62L (d30, Spleen)
gag-specific in G0
gag-specific in G1
not gag-specific in G0
not gag-specific in G1
7.66
1.41
83.23
50.89
Counts
CD62L
E
in "narrow" and FSC-A/-H among not gag-specific (Spleen)
in FSC-A-H
in "narrow" FSC-SSC
% in the indicated gate
Time (d)

Fig. S1

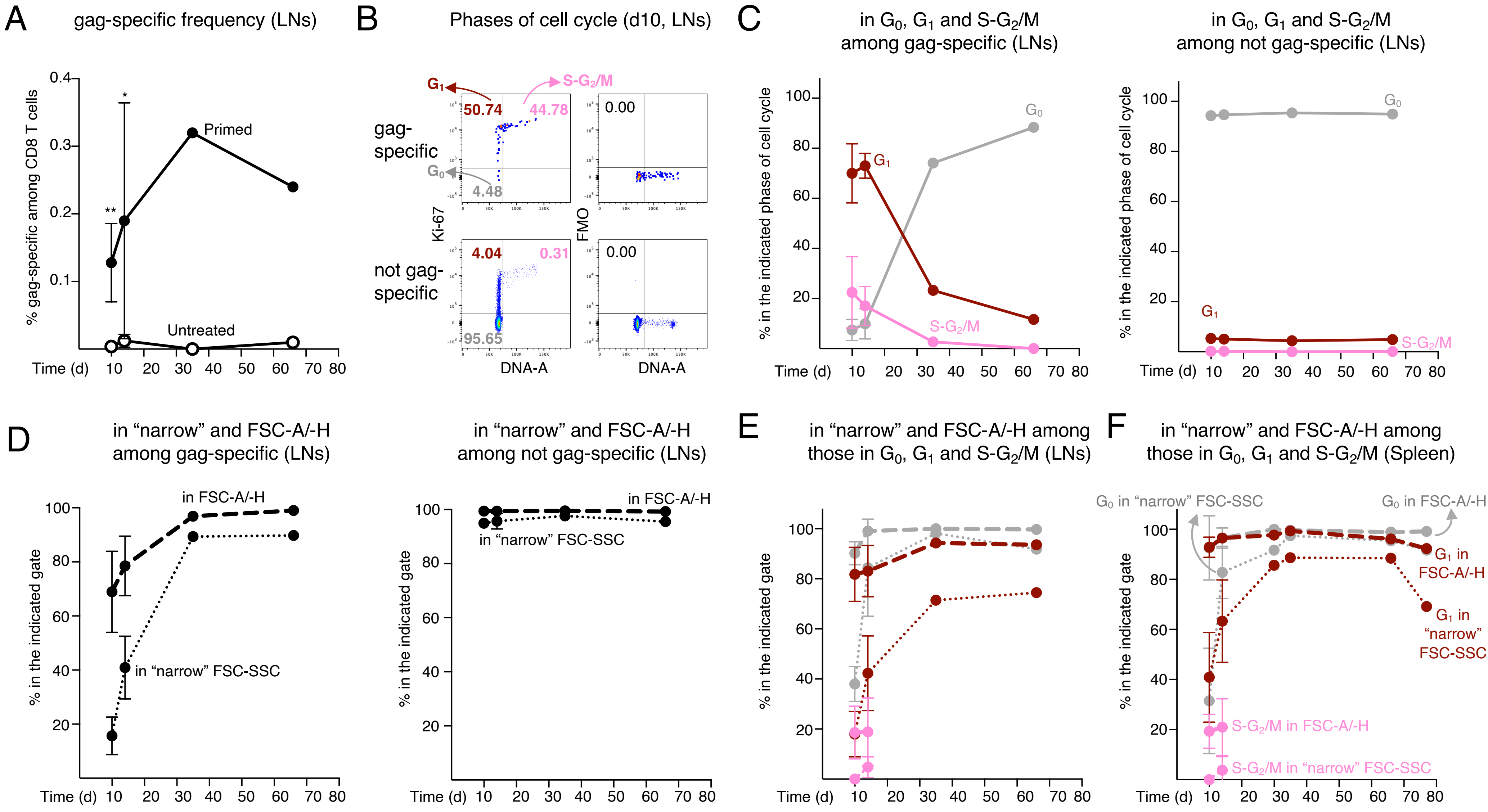

A
gag-specific frequency (LNs)
% gag-specific among CD8 T cells
Primed
Untreated
Time (d)
B
Phases of cell cycle (d10, LNs)
gag-specific
not gag-specific
Ki-67
FMO
DNA-A
50.74
44.78
4.48
0.00
4.04
0.31
95.65
C
in G0, G1 and S-G2/M among gag-specific (LNs)
in G0, G1 and S-G2/M among not gag-specific (LNs)
% in the indicated phase of cell cycle
D
in “narrow” and FSC-A/-H among gag-specific (LNs)
in “narrow” and FSC-A/-H among not gag-specific (LNs)
in FSC-A/-H
in “narrow” FSC-SSC
% in the indicated gate
E
in “narrow” and FSC-A/-H among those in G0, G1 and S-G2/M (LNs)
F
in “narrow” and FSC-A/-H among those in G0, G1 and S-G2/M (Spleen)
G0 in “narrow” FSC-SSC
G0 in FSC-A/-H
G1 in FSC-A/-H
G1 in “narrow” FSC-SSC
S-G2/M in FSC-A/-H
S-G2/M in “narrow” FSC-SSC

Fig. S2

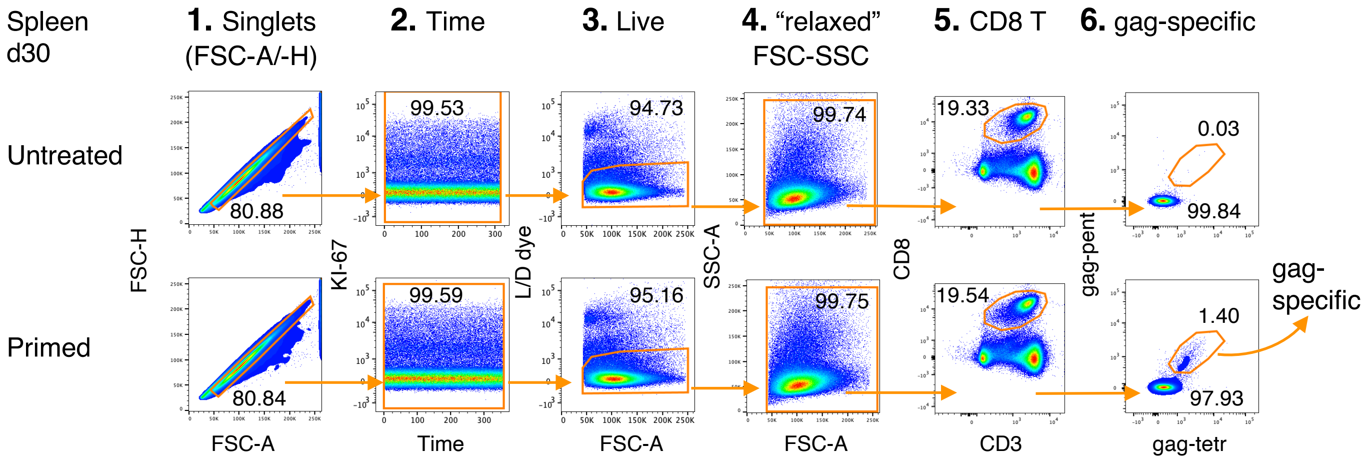

Spleen
d30
1. Singlets
(FSC-A/-H)
2. Time
3. Live
4. "relaxed"
FSC-SSC
5. CD8 T
6. gag-specific
Untreated
Primed
FSC-H
KI-67
L/D dye
SSC-A
CD8
gag-pent
80.88
99.53
94.73
99.74
19.33
0.03
99.84
80.84
99.59
95.16
99.75
19.54
1.40
97.93
gag-
specific
FSC-A
Time
FSC-A
FSC-A
CD3
gag-tetr

Fig. S3

Fig. S4

A

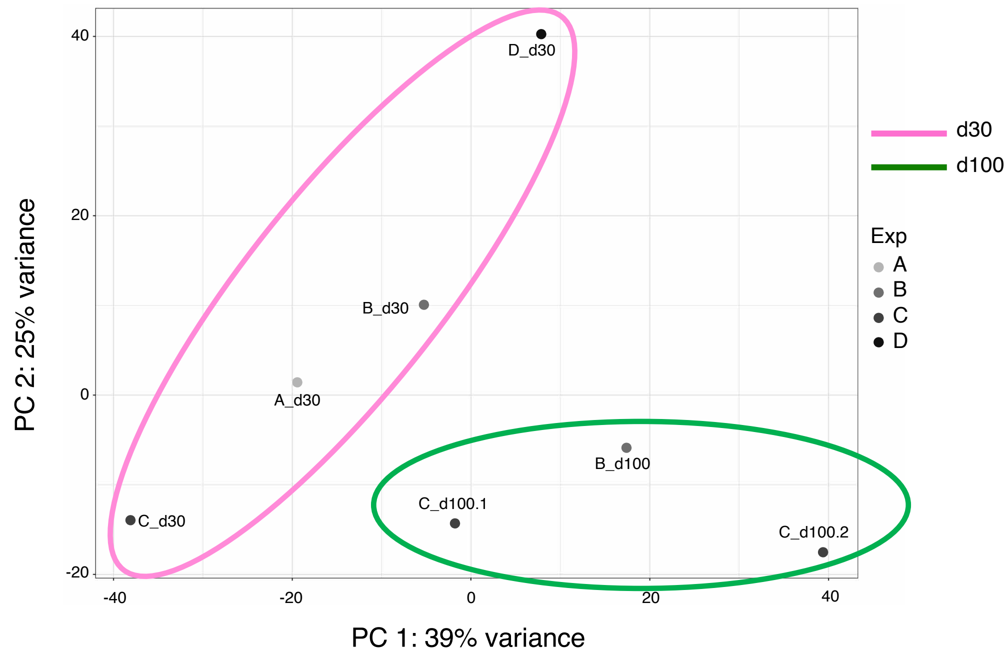

B

variance-stabilised DESeq2 normalized counts, represented using d30 average as the baseline (log2 scale)

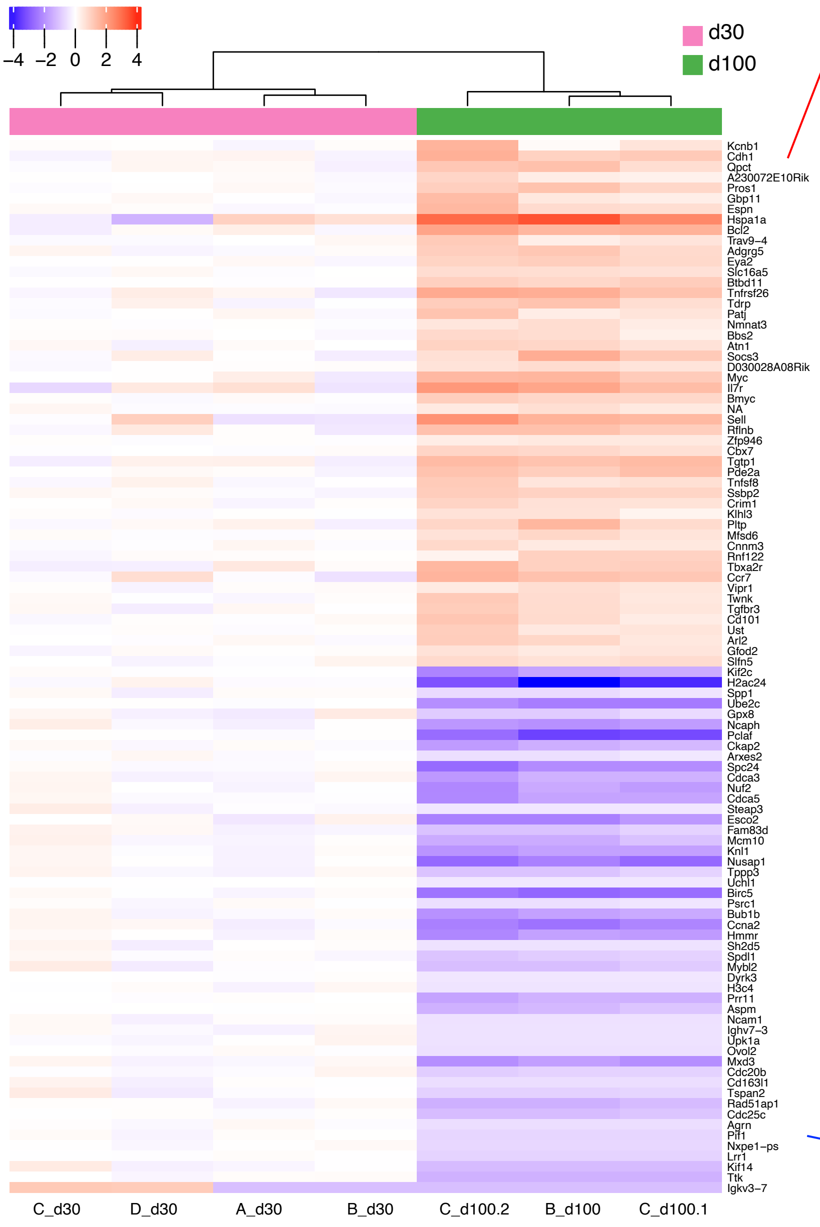

C

TOP 50-UP	LFC
Kcnb1	4.01
Cdh1	3.69
**Qpct**	**2.83**
A230072E10Rik	2.82
**Pros1**	**2.63**
Gbp11	2.46
Espn	2.34
Hspa1a	2.26
Bcl2	2.05
Trav9-4	2.05
Adgrg5	1.98
Eya2	1.92
**Slc16a5**	**1.87**
Btbd11	1.81
Tnfrsf26	1.79
Tdrp	1.78
Patj	1.76
**Nmnat3**	**1.73**
Bbs2	1.70
Atn1	1.68
**Socs3**	**1.67**
D030028A08Rik	1.65
**Myc**	**1.64**
Il7r	1.63
Bmyc	1.57
ENSMUSG00000116180	1.54
Sell	1.54
Rflnb	1.53
Zfp946	1.52
Cbx7	1.52
Tgtp1	1.52
Pde2a	1.51
Tnfsf8	1.51
**Ssbp2**	**1.45**
Crim1	1.43
Klhl3	1.42
Pltp	1.41
Mfsd6	1.38
Cnnm3	1.37
Rnf122	1.35
Tbxa2r	1.33
Ccr7	1.28
Vipr1	1.28
Twnk	1.27
Tgfbr3	1.25
Cd101	1.25
Ust	1.25
Arl2	1.21
**Gfod2**	**1.20**
**Slfn5**	**1.19**

TOP 50-DOWN	LFC
**Kif2c**	**-4.02**
H2ac24	-4.11
Spp1	-4.15
Ube2c	-4.21
Gpx8	-4.35
**Ncaph**	**-4.35**
**Pclaf**	**-4.39**
Ckap2	-4.47
Arxes2	-4.49
**Spc24**	**-4.50**
**Cdca3**	**-4.53**
**Nuf2**	**-4.62**
**Cdca5**	**-4.62**
Steap3	-4.67
**Esco2**	**-4.71**
**Fam83d**	**-4.73**
**Mcm10**	**-4.75**
**Knl1**	**-4.80**
**Nusap1**	**-4.82**
Tppp3	-4.91
Uchl1	-4.94
Birc5	-4.97
**Psrc1**	**-5.09**
**Bub1b**	**-5.11**
**Ccna2**	**-5.14**
Hmmr	-5.27
Sh2d5	-5.33
**Spdl1**	**-5.35**
Mybl2	-5.37
Dyrk3	-5.51
H3c4	-5.77
**Prr11**	**-5.84**
**Aspm**	**-5.94**
Ncam1	-6.03
Ighv7-3	-6.13
Upk1a	-6.15
Ovol2	-6.27
Mxd3	-6.27
**Cdc20b**	**-6.41**
Cd163l1	-6.65
Tspan2	-6.70
Rad51ap1	-6.80
**Cdc25c**	**-6.81**
Agrn	-7.43
Pif1	-7.77
Nxpe1-ps	-7.91
Lrr1	-8.10
**Kif14**	**-9.25**
**Ttk**	**-10.01**
Igkv3-7	-23.56

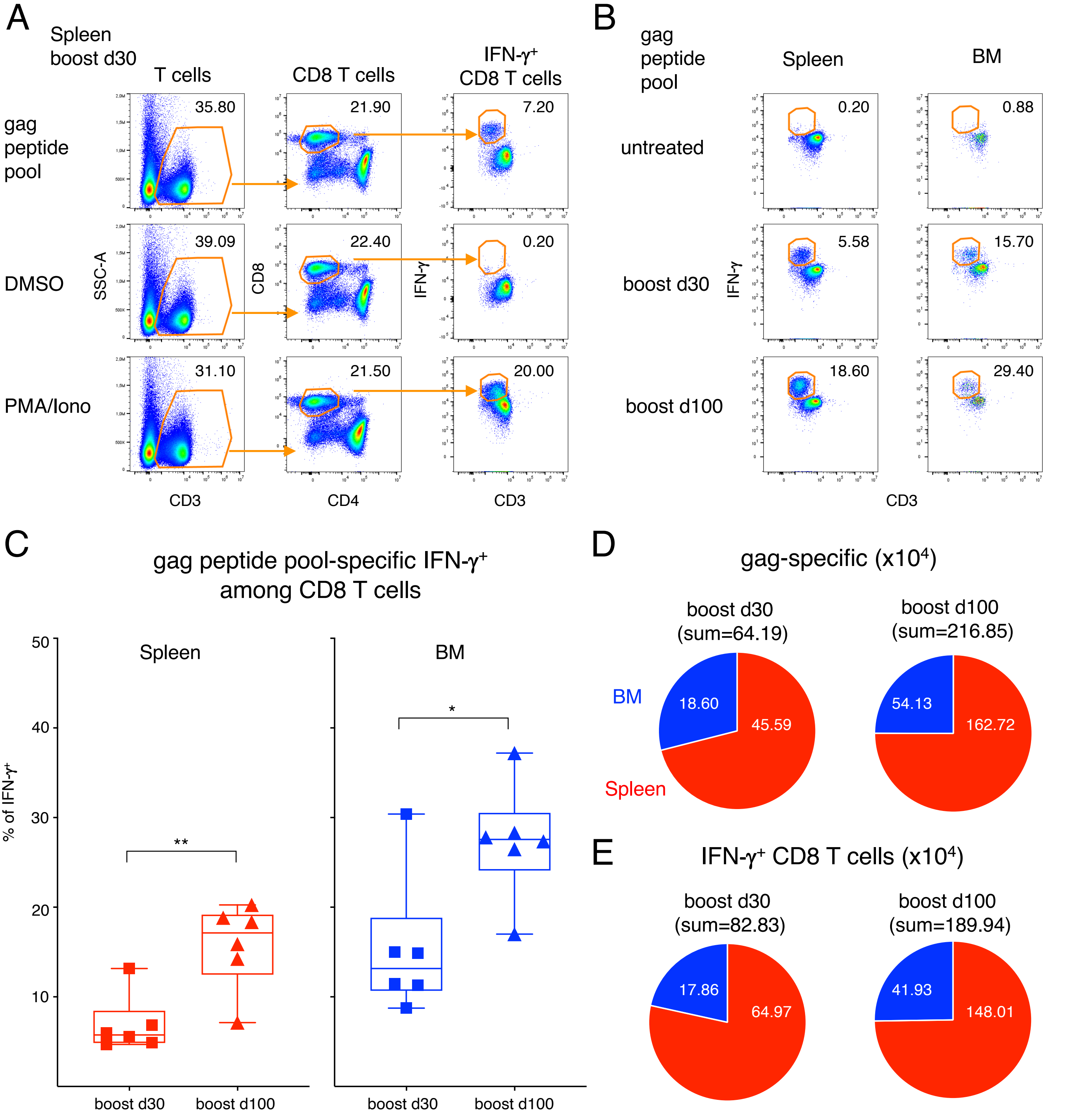
A
Spleen
boost d30
T cells
CD8 T cells
IFN-γ+
CD8 T cells
gag
peptide
pool
DMSO
PMA/Iono
35.80
21.90
7.20
39.09
22.40
0.20
31.10
21.50
20.00
SSC-A
CD8
IFN-γ
CD3
CD4
CD3
B
gag
peptide
pool
Spleen
BM
untreated
boost d30
boost d100
0.20
0.88
5.58
15.70
18.60
29.40
IFN-γ
CD3
C
gag peptide pool-specific IFN-γ+
among CD8 T cells
% of IFN-γ+
Spleen
BM
**
*
boost d30
boost d100
boost d30
boost d100
D
gag-specific (x10^4)
boost d30
(sum=64.19)
boost d100
(sum=216.85)
BM
Spleen
18.60
45.59
54.13
162.72
E
IFN-γ+ CD8 T cells (x10^4)
boost d30
(sum=82.83)
boost d100
(sum=189.94)
17.86
64.97
41.93
148.01

Fig. S5

Supplementary figure legends

Figure S1

Frequency and cell cycle analysis of gag-specific CD8 T cells from ChAd-gag-primed mice: gating strategies for flow cytometry analysis, spleen gag-specific cell phenotype and not gag-specific cell controls.

(A). Membrane and Ki-67/DNA-stained sample gating strategy. Example of gating strategy for flow cytometry analysis of spleen (top) and LN cells (bottom) in 7 steps (gates are indicated in orange, see also ref. (28, 29)): 1) singlet identification based on DNA content; 2) time exclusion, to eliminate any events collected in case of pressure fluctuations; 3) live cell gate; 4) "relaxed" FSC-A/SSC-A (FSC-SSC) gate (the commonly used "narrow" FSC-SSC gate for lymphocytes is shown in white for comparison); 5) CD8 T cell gate; 6) fine exclusion of residual doublets; 7) gag-specific gate, to identify "gag-specific" cells; "not gag-specific" cells were also gated and examined for comparison. Note that we gated out $CD3^{-}$ cells when acquiring spleen samples (step 5, top), and used a stringent criterion for time exclusion to avoid any impact of pressure fluctuations on cell cycle analysis (step 2, bottom). **(B).** Summary of cell cycle phases of not gag-specific CD8 T cells from the primed mice shown in Fig. 1D. (**C-D).** CD127 **(C)** and CD62L **(D)** histogram profiles of gag-specific (top) and not gag-specific (bottom) CD8 T cells from the spleen of primed mice at d30 post-prime. Cells in G_0 (left) and in G_1 (right) were separately analyzed. In **C** numbers represent CD127 Median Fluorescence Intensity, in **D** numbers represent percentages of cells in the indicated regions. **(E).** Kinetics of the percentages of not gag-specific CD8 T cells in the "narrow" FSC-SSC gate and in the FSC-A/-H gate from the primed mice shown in Fig. 1G. Statistical analysis was performed using Wilcoxon test for comparison between not gag-specific CD8 T cells in the "narrow" FSC-SSC gate and in the FSC-A/-H gate at d10 and d14 **(E)**.

Figure S2

Frequency and cell cycle analysis of gag-specific CD8 T cells from ChAd-gag-primed mice: LN and spleen results.

(A-D). LN results. Frequency and cell cycle of LN cells from the primed mice represented in Fig. 1. **(A)**. Summary of the kinetics of gag-specific frequency in LNs of primed and untreated mice. **(B)**. Cell cycle of gag-specific (top) and not gag-specific (bottom) LN cells, evaluated on DNA/Ki-67 plots as in Fig. 1B. **(C)**. Summary of the kinetics of cell cycle phases of gag-specific (left) and not gag-specific (right) CD8 T cells from LNs of primed mice. **(D)**. Kinetics of the percentages of LN gag-specific (left) and not gag-specific (right) CD8 T cells in the "narrow" FSC-SSC gate and in the FSC-A/-H gate **(E-F). Impact of LN and spleen cell gating on detection of cell cycle phases**. Kinetics of the percentages of LN **(E)** and spleen **(F)** gag-specific CD8 T cells in G_0, G_1, and S-G_2/M comprised within either the "narrow" FSC-SSC or the FSC-A/-H gate, as indicated. In **B** numbers represent percentages of cells in the indicated regions. Statistical analysis was performed using Mann-Whitney test for comparison between untreated and primed mice at d10 and d14 **(A)**, and Wilcoxon test for comparison between cells in the "narrow" FSC-SSC gate and their counterparts in the FSC-A/-H gate at d10 and d14 **(D-F)**. Statistically significant differences are indicated (* $P \leq 0.05$; ** $P \leq 0.01$).

Figure S3

Membrane- and Ki-67-stained sample gating strategy for analysis of gag-specific CD8 T cells from ChAd-gag-primed mice at d30, d60 and d100 post-prime

Example of gating strategy for flow cytometry analysis of spleen cells from untreated (top) and primed (bottom) mice in 6 steps: 1) singlet identification based on FSC-A/-H; 2) time exclusion, to eliminate any events collected in case of pressure fluctuations; 3) live cell gate; 4) "relaxed" FSC-A/SSC-A (FSC-SSC) gate; 5) CD8 T cell gate; 6) gag-specific gate, to identify "gag-specific" cells. Numbers represent percentages of cells in the indicated regions. A similar strategy was used for LN, BM and blood cell analysis.

Figure S4

Principal Component Analysis (PCA), and heatmap of the top 50-up and top 50-down statistically significant DEGs.

Bulk RNAseq and bioinformatic analysis were performed as described in Fig. 4 legend and in materials and methods**. (A).** PCA plot representing samples' variance-stabilised normalised counts projected onto the first two principal components. **(B).** Heatmap of the top 50 significantly upregulated (top 50-up) and of the top 50 significantly downregulated (top 50-down) DEGs, in order of their regularized log2 fold-change estimate (LFC); color gradient represents variance-stabilised DESeq2 normalised counts, using the mean of the d30 samples as baseline **(C).** List of the top 50-up and of the top 50-down statistically significant DEGs, ordered as in **B**. For each DEG the corresponding LFC is indicated. In the top 50-up list, genes regulating quiescence and metabolism are highlighted in bold red and bold black, respectively. In the top 50-down list, genes involved in proliferation (DNA replication, mitosis, cell cycle) are highlighted in bold blue. Please note that *Igkv3-7* expression (LFC -23.56) was found only in 2 out of 4 d30 samples, likely reflecting a B cell contaminant, and in 0 out of 3 d100 samples.

Figure S5

Analysis of intracellular IFN-γ production by gag peptide pool-stimulated CD8 T cells at d45 post-boost.

Spleen and BM cells from primed/boosted mice from 2 of the 5 experiments represented in Fig. 5A-B were analyzed at d45 post-boost for intracellular IFN-γ production, after stimulation with either gag peptide pool or its diluent DMSO as negative control. In parallel, cells were also stimulated with PMA/Iono as positive control. **(A).** Example of gating strategy for analysis of spleen cells from mice boosted at d30, after incubation with gag peptide pool (top), DMSO (middle), and PMA/Iono (bottom). Live single cells were gated according to the first 3 steps of fig. S3. Then $CD3^+$ cells were gated on a SSC-A/CD3 plot (left), $CD8^+CD4^-$ cells on a CD4/CD8 plot (center), and IFN-γ^+ cells on a CD3/IFN-γ plot (right). **(B-C).** Examples of CD3/IFN-γ plots representing spleen (left) and BM cells (right) from untreated (top), and primed mice boosted at d30 (middle) and at d100 (bottom) **(B)**, and summary of gag peptide pool-specific IFN-γ^+ CD8 T cell percentages obtained after subtraction of DMSO background **(C)**. **(D-E).** Absolute numbers of gag-specific CD8 T cells **(D)**, and of IFN-γ^+ CD8 T cells **(E)** in spleen and BM at d45 after boost performed at either d30 or d100 post-prime, as indicated. In **A** and **B**, numbers represent percentages of cells in the indicated regions. Panels **C-E** summarize results of 2 independent prime/boost experiments with a total of 18 mice, including control mice (see example in panel **B**). Statistical analysis was performed by Mann-Whitney test. Statistically significant differences are indicated (* $P \leq 0.05$; ** $P \leq 0.01$).
